# A role of oligodendrocytes in information processing

**DOI:** 10.1038/s41467-020-19152-7

**Published:** 2020-10-30

**Authors:** Sharlen Moore, Martin Meschkat, Torben Ruhwedel, Andrea Trevisiol, Iva D. Tzvetanova, Arne Battefeld, Kathrin Kusch, Maarten H. P. Kole, Nicola Strenzke, Wiebke Möbius, Livia de Hoz, Klaus-Armin Nave

**Affiliations:** 1 Department of Neurogenetics, Max Planck Institute of Experimental Medicine, Göttingen, Germany; 2 International Max Planck Research School for Neurosciences, Göttingen, Germany; 3 Göttingen Graduate Center for Neurosciences, Biophysics and Molecular Biosciences, Georg-August-Universität Göttingen, Göttingen, Germany; 4 Center for Nanoscale Microscopy and Molecular Physiology of the Brain, Göttingen, Germany; 5 Department of Axonal Signaling, Netherlands Institute for Neurosciences, Royal Netherlands Academy of Arts and Science, Amsterdam, The Netherlands; 6 Cell Biology, Neurobiology and Biophysics, Department of Biology, Faculty of Science, University of Utrecht, Utrecht, The Netherlands; 7 Institute for Auditory Neuroscience, University Medical Center, Göttingen, Germany; 8 Charité Medical University, Neuroscience Research Center, Berlin, Germany; 9 Present Address: Department of Psychological and Brain Sciences, Krieger School of Arts and Sciences, Johns Hopkins University, Baltimore, USA; 10 Present Address: Sunnybrook Research Institute, Sunnybrook Health Sciences Centre, Toronto, Canada; 11 Present Address: Section of Pharmacology, School of Medicine, European University Cyprus, Nicosia, Cyprus; 12 Present Address: Institut des Maladies Neurodégénératives, Université de Bordeaux, Bordeaux, France

**Keywords:** Cortex, Oligodendrocyte, Neural circuits

## Abstract

Myelinating oligodendrocytes enable fast propagation of action potentials along the ensheathed axons. In addition, oligodendrocytes play diverse non-canonical roles including axonal metabolic support and activity-dependent myelination. An open question remains whether myelination also contributes to information processing in addition to speeding up conduction velocity. Here, we analyze the role of myelin in auditory information processing using paradigms that are also good predictors of speech understanding in humans. We compare mice with different degrees of dysmyelination using acute multiunit recordings in the auditory cortex, in combination with behavioral readouts. We find complex alterations of neuronal responses that reflect fatigue and temporal acuity deficits. We observe partially discriminable but similar deficits in well myelinated mice in which glial cells cannot fully support axons metabolically. We suggest a model in which myelination contributes to sustained stimulus perception in temporally complex paradigms, with a role of metabolically active oligodendrocytes in cortical information processing.

## Abstract (web summary)

Oligodendrocytes myelinate and metabolically support axons. The role of myelination in information processing beyond regulation of conduction velocity is unclear. Here, the authors show that myelination contributes to sustained stimulus perception in the auditory cortex, shaping neuronal responses.

## Introduction

In the central nervous system (CNS), oligodendrocytes assemble myelin, a multilayered sheath of membrane, spirally wrapped around axonal segments and best known for its role in enabling fast saltatory impulse propagation^1,2^. An additional function of oligodendrocytes is the metabolic support of myelinated axons^3–5^, most important when axons spike at high frequencies^6^. Subtle defects of CNS myelin have been associated with various psychiatric diseases^7^, and the aging brain displays subtle but widespread structural deterioration of myelin^8,9^. Basic research has mostly focused on rodent models with white matter abnormalities and analyses largely restricted to the diagnosis and assessment of reduced motor functions. These studies revealed, among other findings, that oligodendrocytes can myelinate axons in an activity-dependent manner and influence, as predicted, their conduction velocity properties^10,11^. In a highly specialized brainstem circuit, the thickness and length of axonal internodes are critical for impulse propagation with submillisecond precision, and nerve conduction velocity is fine-tuned by myelination^12,13^, all of this required for sound localization^14^. Interestingly, in the cortex, myelin is sparse^15–17^, and it is not clear how changes in myelination would affect conduction and function in the intracortical circuitry. Also, while a large fraction of cortical parvalbumin-positive interneuron axons is myelinated^16,18^, they typically show thin axons and short path lengths to their target cells. Similarly, the small caliber axons in the cingulate cortex conduct at slow speed, despite being myelinated^19^. These data raise the question whether the function of myelin goes beyond the regulation of conduction velocity. We note that mature oligodendrocytes respond to glutamatergic signals with enhanced glycolytic support of the axonal energy metabolism^5,6^.

At the network level, investigating the contributions of CNS myelination to information processing has been challenging because changes in myelin lead to changes in axonal properties. Moreover, suitable phenotyping instruments are limited. We assume that the function of oligodendrocytes should be most evident for pathways that build on constant information transfer, as it is the case for the auditory system. In animal models of CNS dysmyelination, a few studies pointed at general auditory abnormalities^20–23^ or a specific delay at central auditory stations^24–26^, detectable by increased response latencies. In the brainstem sound localization circuit, upon dysmyelination, excitability defects as well as increased jitter have been reported^23^. However, the perceptual consequences of these deficits are not understood. Moreover, the sound localization circuit is highly specialized and dedicated to submillisecond precision of coincidence detection. Auditory processing is clearly broader than sound localization and must constantly cope with the appearance of both short and repetitive stimuli. This is essential, for example, for speech perception. In humans, myelin abnormalities and nerve conduction velocities have been mostly studied in terms of developmental delays, and rarely with respect to signal detection, precision, and acuity. Yet, it is clinically well known that patients with myelin diseases, similar to individuals of old age, show deficits in auditory processing^27,28^ that might have more complex explanations than abnormal cochlear function. For example, deficits in speech recognition in noisy environments can be present, despite normal hearing thresholds^29^, which is suggestive of problems in temporal acuity in higher-order centers, rather than cochlear dysfunction.

To our knowledge, there is no in vivo evidence for a role of myelin in sensory perception. Here, we tested the hypothesis that the dual role of myelinating oligodendrocytes in speeding impulse propagation and providing metabolic support to the spiking axon contributes to information processing in the CNS and can be experimentally analyzed in the auditory system of mice. We aimed to determine how dysmyelination and/or impairments in axoglial metabolic support affect temporal and spectral auditory processing. The tight axon–myelin interaction trammels the study of the multiple individual roles that oligodendrocytes might be playing in signal processing since, for example, the absence of myelin radically changes axon calibers^30^. For this reason, we generated a new mouse model with partial myelin loss but normal axon caliber distribution (*Mbp*^*neo/neo*^). In this study, we compared adult wild-type (Wt) animals with three lines of mutant mice: (1) the shiverer mice (*Mbp*^*shi/shi*^)^31,32^ as a model of severe dysmyelination, (2) a newly generated *Mbp* allele (*Mbp*^*neo/neo*^) as a hypomyelination model (see Supplementary Fig. S1), and (3) mice with the heterozygous null allele of the *Slc16A1* gene (*Mct1*^*+/−*^), encoding a monocarboxylate transporter (MCT1) that is required for glial metabolic support^4^. Collectively, these mutants allowed the comparison of different degrees of myelination (models 1 and 2), and to specifically assess the role of glial cells in axonal metabolic support (model 3).

We tested auditory processing in mice using cortical multiunit recordings and behavioral readouts. Our data reveal that myelinating oligodendrocytes determine the quality of information processing, and suggest that this might be partially dependent on their metabolic support functions.

## Results

To monitor auditory processing in the adult brain with compromised oligodendrocyte functions, we used three types of mutant mice. To study the role of compact myelin, we compared two different *Mbp* mutant mouse lines. MBP is an abundant structural protein required for myelin growth and compaction^33,34^. One mutant is defined by the nearly complete absence of CNS myelin, the well-known shiverer (*Mbp*^*shi/shi*^) mouse^31,32^ with a truncated *Mbp* gene^31^. The second *Mbp* model was newly generated for this study as a hypomorph mouse (*Mbp*^*neo/neo*^) with MBP expression levels below 50% as detailed below. To further understand the deficit in auditory processing observed in the *Mbp* mutants, we used an additional mouse model that is myelinated but exhibits deficits in the oligodendrocytes’ metabolic support of the axon^4^. This mutant, heterozygous null for the Slc16A1 gene (*Mct1*^*+/−*^), shows reduced expression of the monocarboxylate transporter MCT1, which is required by oligodendrocytes and astrocytes to metabolically support axons^4^. MCT1 enables the export of lactate and pyruvate, and supports the generation of ATP in axons^6^. While MCT1 is expressed in oligodendrocytes in the CNS^4^, it is also expressed in astrocytes and endothelial cells^35^.

### *Mbp*^*neo*^: a novel mouse mutant with reduced myelin sheath thickness

Shiverer mice lack compact myelin and display a shivering phenotype that makes them suboptimal for behavioral testing. Since heterozygous *Mbp*^*shi/+*^ mice are well myelinated^36^, we sought to establish a new hypomyelinated mouse model by reducing *Mbp* expression levels below 50% (see Methods and Supplementary Fig. S1A), in agreement with earlier observations of transgenic complementation of shiverer mice^37,38^. Oligodendrocytes in adult *Mbp*^*neo/neo*^ brains expressed 30% of the *Mbp* mRNA (Supplementary Fig. S1B) and 20% MBP at the protein level (Supplementary Fig. S1C). These mutants appeared clinically normal and were long-lived. Unlike conventional shiverer mice, in which the vast majority of optic nerve axons are unmyelinated^32^, *Mbp*^*neo/neo*^ mice have 75% of their optic nerve axons myelinated (Wt: 92%). Importantly, this myelin was fully compacted but significantly thinner, as determined by g-ratio analysis (Supplementary Fig. S1D–F). An essential feature of this mouse model is that axon caliber distribution was not different between Wt and *Mbp*^*neo/neo*^ mice (Supplementary Fig. S1G).

### Myelination of the primary auditory cortex is patchy and heterogeneous

**Fig. 1:**
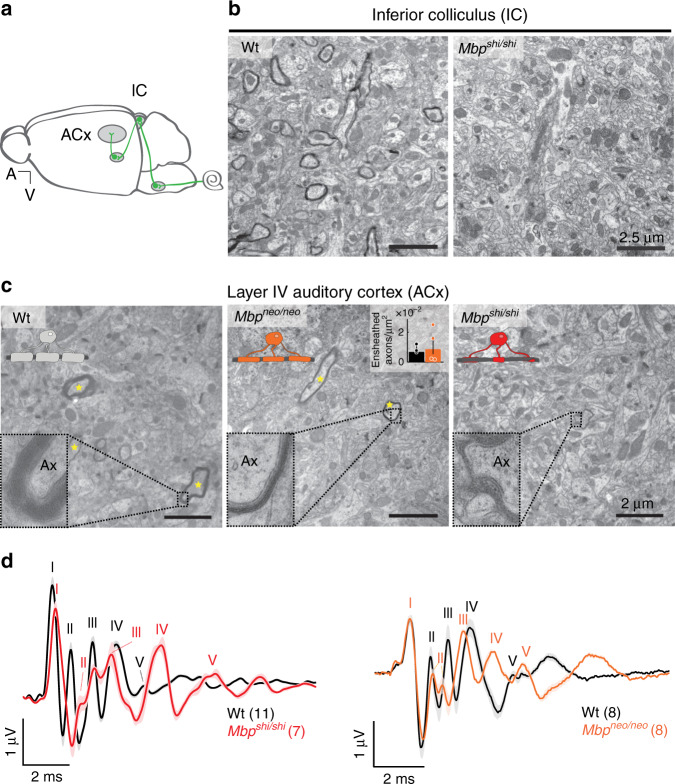
Ensheathment profiles along the auditory system in normal and dysmyelinated models. **a** Scheme illustrating the auditory pathway with emphasis on the location of the inferior colliculus (IC) and auditory cortex (ACx). **b** Electron microscopy images of the IC of a Wt mouse (left panel) showing sparse compact myelin, and an *Mbp*^*shi/shi*^ mouse (right panel), lacking electro-dense compact myelin. **c** Electron microscopy images of the auditory cortex of Wt (left), *Mbp*^*shi/shi*^ (middle), and *Mbp*^*neo/neo*^ mouse (right). Properly ensheathed axons in the ACx are marked with yellow asterisks. Insets show details of the myelin sheath of axons (Ax) from the respective image. *Mbp*^*shi/shi*^ axons (right) show lack of compact myelin, while *Mbp*^*neo/neo*^ axons (middle) show thinner compact myelin than Wt. The inset plot (middle) shows the quantification of the number of ensheathed axons per area in Wt (black, *n* = 3) and *Mbp*^*neo/neo*^ (orange, *n* = 4), (two-sided Wilcoxon rank-sum test, *P* = 0.73, *t* = 0.434). The bar graph show the mean of all animals quantified (10–15 images per mouse). **d** Auditory brainstem-response (ABR) potentials. Left: group mean traces of control (black, *n* = 11). Pooled together seven *Mbp*^+/+^ and four *Mbp*^*shi/+*^, see Supplementary Fig. S2E for significances, and *Mbp*^*shi/shi*^ (red, *n* = 7) mice. Each one of the five peaks (I–V) can be attributed to activity at a different station along the auditory brainstem (see Supplementary Fig. S2Aii). Responses in *Mbp*^*shi/shi*^ mice were delayed at all auditory stations. Wave II appears divided and merged with wave III. Right: group mean traces of control (black, *n* = 8) and *Mbp*^*neo/neo*^ (orange, *n* = 8) mice. Responses in *Mbp*^*neo/neo*^ mice were delayed at all auditory stations. Scale bars: 2.5 µm (**b**), 2 µm (**c**). Source data are provided as a Source Data file.

To study auditory phenotypes, we first assessed the presence of myelin in the inferior colliculus (IC), an important subcortical relay station in the auditory pathway (Fig. 1a), and in layer IV of the primary auditory cortex (ACx), where we obtained multiunit recordings. As in other gray matter areas, myelination profiles are present at low density^15,16^. Compared to the IC (Fig. 1b), myelination of the ACx was even sparser (Fig. 1c, left panel). While Wt axons were surrounded by electron-dense (compact) myelin (Fig. 1c, left panel), *Mbp*^*shi/shi*^ axons were loosely ensheathed with uncompact myelin (Fig. 1c, right panel) and *Mbp*^*neo/neo*^ mice exhibited an intermediate profile with compact but thinner myelin compared to Wt (Fig. 1c, middle panel). Dysmyelination of *Mbp*^*neo/neo*^ was obvious by electron microscopy (Fig. 1c mid panel and inset; Supplementary Fig. S1D). Interestingly, when quantified, *Mbp*^*neo/neo*^ mice had the same number of ensheathed axons in the ACx as their respective controls (Fig. 1c, middle-panel graph).

### Central dysmyelination causes signs of auditory neuropathy

In Wt mice, all auditory projections, including the auditory nerve, are well myelinated^39,40^. Before studying the effect of dysmyelination on information processing, we determined latencies and amplitudes of sound-evoked responses at different auditory stations using auditory brainstem responses (ABR), recorded in vivo through electrodes located over the scalp (Supplementary Fig. S2Ai). ABRs consist of five waves (I–V) that reflect the consecutive activation of the auditory nerve, cochlear nucleus, superior olive, lateral lemniscus, and inferior colliculus (Supplementary Fig. S2Aii). In the *Mbp*^*shi/shi*^ mice, response thresholds were not different from controls (Supplementary Fig. S2B, C). ABR waves were however significantly delayed at all central auditory stations (Fig. 1d left and Supplementary Fig. S2H) and delays increased progressively. The peak-to-peak amplitude, which reflects the synchronous spiking in response to sound onset, was unchanged in wave I (Fig. 1d and Supplementary Fig. S2I). This, together with the finding of unchanged thresholds, confirms that cochlear function and peripheral conduction is normal in *Mbp*^*shi/shi*^ mice. This was expected because the outer region of the auditory nerve is myelinated by Schwann cells that do not require MBP to form compact myelin (Supplementary Fig. S2Aiii)^40^. However, the amplitudes of waves II and III tended to be reduced, and those of waves IV and V slightly increased (Fig. 1d left and Supplementary Fig. S2I). Such downstream compensation of diminished brainstem ABR amplitudes has been observed in several animal models of auditory synaptopathy/neuropathy (disorders of the inner hair cell ribbon synapse and/or auditory nerve), and is thought to reflect the compensatory mechanism of changes in gain to peripheral hearing loss^41–46^. Hypomyelinated *Mbp*^*neo/neo*^ mice were similar to Wt with respect to the shape of ABR wave I (Fig. 1d right) and hearing thresholds (Supplementary Fig. S2D). They also showed an increase in latency (Fig. 1d right and Supplementary Fig. S2F) and a significant reduction in wave III amplitude, as well as an amplitude compensation in wave V (IC level) (Supplementary Fig. S2G), indicating that even mild central dysmyelination can cause signs of auditory neuropathy.

### Dysmyelination affects temporal reliability and acuity of sound processing

Naturally occurring sounds are characterized by rich temporal structures. In many species, the reliable temporal coding of communication sounds is an important factor for survival. Correct auditory coding relies on the ability of the nervous system to convey the temporal structure of sounds with precise spike timing and to maintain this precision while listening to continuous sound streams. To test the role of myelin and oligodendrocytes in temporal processing, we moved from scalp recordings to acute in vivo extracellular recordings of neuronal spikes using tungsten electrodes placed in layer IV of the primary auditory cortex. We determined, on one hand, the neuronal capacity of our mouse models to follow click trains (a measure of temporal reliability) at different presentation rates. We also applied a gap-detection paradigm to measure temporal acuity, similarly used for audiometric testing in humans^47–49^.

**Fig. 2:**
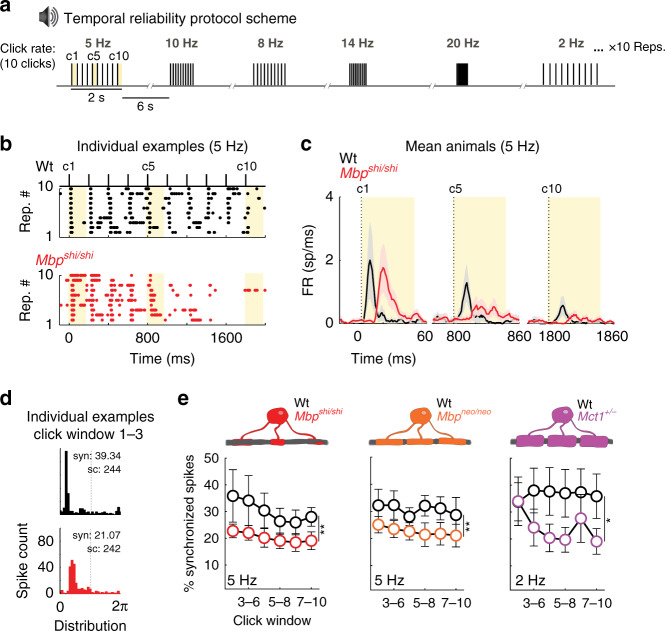
Temporal reliability is affected in mice with either dysmyelination or an oligodendrocyte-specific metabolic impairment. **a** Schematic of the click-rate-tracing protocol used to test temporal reliability. Blocks of ten clicks are played at different rates in random order. Each rate is repeated ten times. Analysis in **c** focuses on the highlighted (yellow) clicks 1, 5, and 10 (c1, c5, and c10). **b** Example raster plot of ACx responses (each dot = 1 spike) to ten clicks at 5 Hz across the ten stimulus repetitions in a Wt (upper, black) and an *Mbp*^*shi/shi*^ mouse (lower, red). *Mbp*^*shi/shi*^ animals show a steeper decay on spiking activity across clicks compared to Wt. **c** Mean peristimulus time histogram (PSTH) of responses to clicks 1, 5, and 10, at 5 Hz for Wt (black, *n* = 10) and *Mbp*^*shi/shi*^ (red, *n* = 13) animals. The thick line shows the mean of all recorded animals and the shaded area depicts the S.E.M. Click onset is indicated by dashed lines. While responses to the first click are similar in amplitude in Wt and *Mbp*^*shi/shi*^ animals (with the expected delay in *Mbp*^*shi/shi*^), a strong reduction of response strength is seen in *Mbp*^*shi/shi*^ mice with increasing clicks. **d** Individual examples of spike synchrony plots for Wt (black, top) and *Mbp*^*shi/shi*^ (red, bottom) were taken from the first sliding window (see panel **e** clicks 2–5). Syn: synchrony %, sc: spike count. **e** Quantification of spike synchrony in sliding windows of four clicks (clicks 2–5, 3–6, 4–7, 5–8, 6–9, and 7–10). Onset responses to click 1 were excluded. Leftmost: significant reduction in spike synchrony between *Mbp*^*shi/shi*^ mice (red, *n* = 11) and Wt (black, *n* = 5 mice) at 5 Hz (one-way ANOVA, *F*(1,76) = 10.17, *P* = 0.002). Middle: significant reduction also seen between *Mbp*^*neo/neo*^ mice (orange, *n* = 6 mice) and Wt (black, *n* = 8 mice) at 5 Hz (one-way ANOVA, *F*(1,86) = 10.94, *P* = 0.0014). Right: significant difference between *Mct1*^*+/−*^ mice (purple, *n* = 6 mice) and Wt (black, *n* = 8 mice) at 2 Hz (one-way ANOVA, *F*(1,78) = 6.74, *P* = 0.011). Source data are provided as a Source Data file.

In Wt mice, the rates at which auditory neurons can follow were lower in ACx compared to subcortical stations, consistent with previous reports^50^. Click-rate detection was tested with sets of ten clicks presented at different temporal rates (Fig. 2a). Repeated presentations of sets of ten clicks revealed that the timing of spikes is reproducible from set to set, although failures were observed in the response to the last click in a set (example Fig. 2b, top). Importantly, in *Mbp*^*shi/shi*^ mice, ACx neurons followed the initial five clicks in a set of ten, but (at 5 Hz) response strength to the last click strongly decayed (example Fig. 2b, bottom). Scoring the responses to click numbers 1, 5, and 10 (taken as representatives), we observed that the response magnitude in *Mbp*^*shi/shi*^ mice was the same as in Wt after click 1, but was smaller after click 5 and virtually nonexistent by click 10 (Fig. 2c). This decrease in response magnitude was associated with a decreased synchrony of spiking (across repetitions along the 10 clicks) compared to Wt (Fig. 2d) for both repetitions, at rates of 8 Hz (Supplementary Fig. S3A) and 5 Hz (Fig. 2e, left). A similar effect was observed in the cortex of *Mbp*^*neo/neo*^ mice (Supplementary Fig. S3B and Fig. 2e, center). Thus, naked axons of *Mbp*^*shi/shi*^ mice were not a prerequisite for decreased synchrony. A similar but weaker effect was evident also at slower click rates in the *Mct1*^*+/−*^ mutants with reduced expression of the monocarboxylate transporter (Fig. 2e, right and Supplementary Fig. S3C). Here, the effect was evident at 2 Hz. At higher rates, the Wt mice show unusually lower synchrony, as do the mutants, maybe unveiling a floor effect. Interestingly, we did not find any signs of axonal degeneration in 12–16-week-old *Mct1*^*+/−*^ mutant mice (Supplementary Fig. S4A–D), thereby uncoupling axon function defects from the previously reported age-dependent neurodegeneration of these mutant animals^4^. Mutant auditory brainstem responses (ABRs) were not impaired in threshold, amplitude, or latency (Supplementary Fig. S4E–H), supporting the notion that latency increases are a result of partial or complete dysmyelination, unrelated to metabolic defects.

A certain amount of variability in baseline performance was observed among the different mouse lines. Therefore, we always performed within-line comparisons using littermate controls. Wt responses were largely consistent across lines. We found that both dysmyelination (*Mbp*^*shi/shi*^ and *Mbp*^*neo/neo*^) and a partial axoglial metabolic deficit (*Mct1*^*+/−*^) led to cortical auditory fatigue in response to click trains, in comparison with Wt littermates.

Overall, the data suggest that axonal energy deficits in the absence of dysmyelination, caused by perturbed glial lactate export^4^, also cause conduction failure in the auditory pathway when neurons are repetitively firing. In dysmyelinated shiverer mutants, additional factors may contribute to this effect, such as redistributed ion channels and concomitant abnormal energy consumption.

In the *Mbp*^*shi/shi*^ mice, deficits in temporal reliability could be explained by the altered K_v_ channel distribution (dispersed along internodes and overexpressed in *Mbp*^*shi/shi*^) in axonal excitable domains^51^, which we confirmed in the form of shortened AIS for the ACx of *Mbp*^*shi/shi*^ mice (Supplementary Fig. S5A). In a dysmyelinated brain, axonal excitability, membrane repolarization, and energy consumption could be affected by the general misdistribution of ion channels^52,53^. As a model for CNS white matter tracts that are difficult to study directly, we used a well-established optic nerve preparation^54^. This acute ex vivo system (Supplementary Fig. S5B) allowed us to measure compound action potentials (CAP) from myelinated axons (Supplementary Fig. S5C) and to apply pharmacological manipulations^5,6,54^. In Wt optic nerves (ON), CAPs exhibited the expected 3-peak shape, but we observed strong differences with both *Mbp*^*shi/shi*^ and *Mbp*^*neo/neo*^ nerves. In these mutants, CAP peaks were delayed in latency (Supplementary Fig. S5C), reflecting slowed nerve conduction velocity (Supplementary Fig. S5D) proportional to the level of dysmyelination. Dysmyelinated ON also showed a decrease in peak amplitude and CAP area (Supplementary Fig. S5F), suggesting conduction blocks in the ON, and an increase in the hyperpolarizing phase with a larger negative CAP area (Supplementary Fig. S5G). These features were similar to CAP recordings from the spinal cord of *Mbp*^*shi/shi*^ mice^51^. To test our hypothesis that excess potassium fluxes in dysmyelinated *Mbp*^*shi/shi*^ optic nerve axons cause energy loss (due to repolarization-associated ATP consumption) and contribute to reduced temporal reliability, we blocked potassium channels with 4-aminopyridine (4-AP, 25 µM) in a bath application. This treatment normalized the CAP amplitude and decreased the hyperpolarizing phase of *Mbp*^*shi/shi*^ axons (Supplementary Fig. S5E, gray, S3F and S5G, rightmost). This finding is compatible with abnormal ion fluxes in the mutant axons, which increased ATP consumption and abnormal repolarization dynamics.

**Fig. 3:**
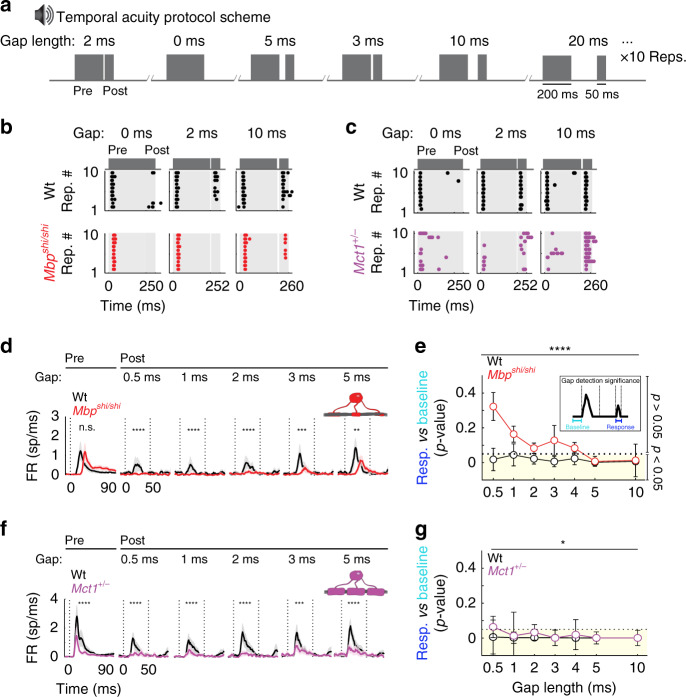
Cortical temporal acuity is severely impaired with central nervous system (CNS) dysmyelination and partially impaired upon axoglial metabolic reduction. **a** Temporal acuity test: gap-detection protocol. A pregap broadband noise (BBN, 200 ms) followed by a silent gap (0–50 ms, ten repetitions each) and post-gap BBN (50 ms). **b** Example for Wt (upper, black) and *Mbp*^*shi/shi*^ (lower, red): sound-evoked (gray patch) ACx spikes (dots) before/after gaps of 0-ms (left), 2-ms (center), and 10-ms (right) length, across ten repetitions. **c** Same as in **b** for the *Mct1* (purple). **d** Average peristimulus time histogram (PSTH) for Wt (black, *n* = 12) and *Mbp*^*shi/shi*^ (red, *n* = 14). Left to right: pregap responses followed by post-gap responses for 0.5–5-ms gaps. Effect of group for post-gap, but not pregap, responses (two-way ANOVAs [*F*(1,462) = 0.53, *P* = 0.46], [*F*(1,502) = 24.47, *P* = 1.03 × 10^−6^], [*F*(1,502) = 20.48, *P* = 7.54 × 10^−6^], [*F*(1,502) = 24.06, *P* = 1.26 × 10^−6^], [*F*(1,502) = 14.38, *P* = 0.0002], and [*F*(1,502) = 7.62, *P* = 0.006]. **e** Quantification of significance (median *p* value between baseline and post-gap response/recording, see inset). Lower detection in *Mbp*^*shi/shi*^ (red, *n* = 20 sites, 14 animals) than Wt (black, *n* = 15 sites, 12 animals) for short gaps, group effect (two-sided Kruskal–Wallis, *F*(1,214) = 15.81, *P* = 7 × 10^−5^). Dotted line: threshold at 0.05. Yellow shadow: significant gap detection. **f** Same as **d** for *Mct1*^*+/−*^ (purple, *n* = 6) and Wt (black, *n* = 8). Group effect for all responses (two-way ANOVAs [*F*(1,252) = 30.06, *P* < 0.0001], [*F*(1, 313) = 24.42, *P* = 1.26 × 10^−6^], [*F*(1,313) = 15.74, *P* = 9.02 × 10^−5^], [*F*(1,313) = 36.65, *P* = 4.03 × 10^−9^], [*F*(1,313) = 10.53, *P* = 0.001], and [*F*(1,313) = 21.3, *P* = 5.73 × 10^−6^]. **g** Same as **e** for *Mct1*^*+/−*^ mice (purple, *n* = 9 sites, six animals) and Wt mice (black, *n* = 13 sites, eight animals). Group effect (two-sided Kruskal–Wallis, *F*(1,151) = 8.83, *P* = 0.0027). **d**, **f** Dotted lines: sound onset and offset. **e**, **g** Circles: median/group/gap length, and error bars: S.E.M. Source data are provided as a Source Data file.

Returning to in vivo electrophysiology of cortical neuron activity, and following the quantification of temporal reliability, we then tested a second key aspect of auditory processing: temporal acuity. Gap-detection protocols measure the ability of neurons to detect short silent gaps in the middle of a white noise sound (Fig. 3a), a capacity also crucial in human speech processing^55^. In the mouse cortex, the presence and the strength of any post-gap sound response, reflects whether the gap has been detected (Fig. 3b). Compared to Wt*, Mbp*^*shi/shi*^ mice revealed a significant decrease in their post-gap response strength for gaps shorter than 3 ms in duration (Fig. 3b, d). Quantification of this effect (by comparing for each recording the ratio between baseline activity and post-gap activity) confirmed that the cortical neurons of *Mbp*^*shi/shi*^ mice failed to detect small gaps (Fig. 3e). A milder effect was observed in *Mbp*^*neo/neo*^ mice (Supplementary Fig. S3D–S3F), suggesting that these temporal acuity deficits reflect the level of dysmyelination. Importantly, *Mct1*^*+/−*^ mice also revealed a gap-detection deficit, albeit minor (Fig. 3c, f, g). Thus, loss of temporal acuity can be influenced by reduced glial metabolic support of the axonal compartment.

### Behavioral correlates of reduced temporal acuity

To understand the correlation between gap-detection deficits and perception at the behavioral level, we performed two behavioral tests. First, we tested gap-dependent prepulse inhibition of the acoustic startle reflex (GDIASR). This paradigm is widely used to behaviorally assess the detection of gaps within sound stimuli^56,57^. For this test, we used hypomyelinated *Mbp*^*neo/neo*^ mice that, unlike the *Mbp*^*shi/shi*^ mice, have no motor defects.

**Fig. 4:**
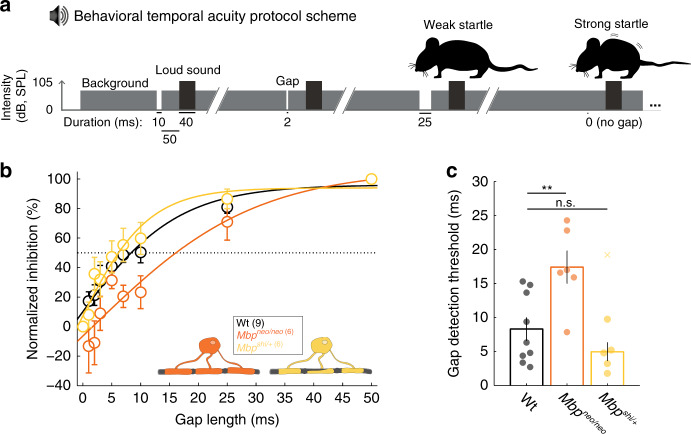
Behavioral temporal acuity is impaired by central nervous system (CNS) dysmyelination. **a** Schematic of the auditory startle reflex (ASR) sound protocol. Constant background broadband noise (BBN, 70 dB) interrupted by a startle noise at random times (105 dB, 40 ms), occasionally preceded by a silent gap of varying length. All gaps presented were followed by 50-ms background sound before the startle appearance. The silent gap, if detected, diminished the startle effect of the loud noise. Each gap-startle combination was repeated ten times. **b** The percentage of ASR inhibition elicited by the different gaps showed a strong relationship between the gap length and the startle inhibition. *Mbp*^*neo/neo*^ (orange, *n* = 6) but not *Mbp*^*shi/+*^ mice (with a 50% reduction in *Mbp*, yellow, *n* = 6) showed impaired inhibition of the ASR. Dotted line: threshold at 50% inhibition used for statistical analysis in **c**. **c** Gap-detection threshold is increased in *Mbp*^*neo/neo*^ mice (two-sided Wilcoxon rank-sum test, *P* = 0.0048, *t* = 3.814), but not *Mbp*^*shi/+*^ (two-sided Wilcoxon rank-sum test, *P* = 0.36, *t* = −2.485; outlier depicted with a cross), compared to Wt (black, *n* = 9) animals. All graphs depict the mean and S.E.M. and individual data points are individual animals. Outliers are depicted with an x and were not considered in the statistical analysis. Source data are provided as a Source Data file.

Prepulse inhibition of the acoustic startle reflex (ASR) requires sensorimotor gating. Acoustic startles are triggered by a loud unexpected sound^58^ and can be measured by a piezo element placed under the animal (Supplementary Fig. S6A). A change in the sound background, for example, a silent gap just before the startling sound (Fig. 4a), can inhibit the ASR if it is salient (long) enough^59^. In this paradigm, we used the level of inhibition by different silent gap lengths (Supplementary Fig. S6B) as a behavioral measure of gap detection (i.e., gap perception). Consistent with previous reports from Wt mice^56,57,59,60^, shorter gaps elicited less inhibition than longer gaps (Fig. 4b). Importantly, hypomyelinated *Mbp*^*neo/neo*^ mice showed a significant decrease in the inhibition of the ASR, when triggered by different gap lengths (Fig. 4b, orange). Moreover, we determined a twofold increase in the gap-detection threshold of *Mbp*^*neo/neo*^ mice (from 8 ms to 17 ms) (Fig. 4c, orange). Thus, deficits in temporal acuity, discovered at the physiological level in the ACx, were also paralleled at the perceptual level. We note that mice with slightly higher levels of *Mbp* expression, i.e., thinly myelinated heterozygous *Mbp*^*shi/+*^ mutants, did not show a reduction in basic startle or in gap perception (Fig. 4b, c, and Supplementary Fig. S6D, yellow). Thus, significant hypomyelination (>50%) is required to elicit gap-detection deficits at the behavioral level.

To confirm altered gap perception in freely behaving mice, we tested *Mbp*^*neo/neo*^ mutants in the AudioBox (New*Behavior*, TSE systems), an automated system for auditory behavioral testing^61–65^. Animals were exposed to safe visits of an enclosed cage corner (Supplementary Fig. S6E), in which a continuous sound was played, and water was always available. However, during conditioned visits in that corner, the continuous sound was interrupted by a series of 50-ms silent gaps that were paired (i.e., negatively conditioned) with short airpuffs to the nose when animals attempted to drink. We then compared the sensitivity to different gap lengths in mutants and controls ranging between 1 and 45 ms (Supplementary Fig. S6F, H). For each gap length, we quantified the percentage of visits in which the mice avoided penalized nosepokes, indicating perception of this gap. Despite equivalent sound exposure in the AudioBox (Supplementary Fig. S6G), gap-detection threshold was increased to 4 ms in *Mbp*^*neo/neo*^ compared to the 2 ms threshold of Wt mice (Supplementary Fig. S6I). We conclude that in freely moving animals, myelination contributes to auditory perceptual acuity.

### Cortical responses to simple sounds in dysmyelinated mice

**Fig. 5:**
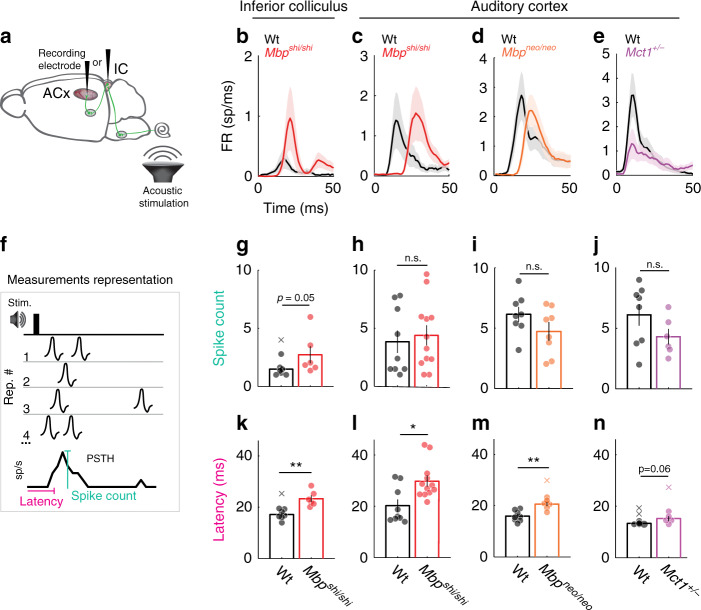
Responses to single sound stimuli in IC and ACx of dysmyelinated models are abnormal only in latency. **a** Recording locations (inferior colliculus or cortex). **b** Peristimulus time histogram (PSTH) of responses to a click sound recorded in the IC for Wt (black, *n* = 6) and *Mbp*^*shi/shi*^ (red, *n* = 7) mice. Delayed *Mbp*^*shi/shi*^ responses. **c**–**e** Same as in **b** but for ACx recordings for *Mbp*^*shi*^, *Mbp*^*neo*^, and *Mct1* lines, respectively. **c** Response delay in *Mbp*^*shi/shi*^ and **d** *Mbp*^*neo/neo*^ mice (orange), and decrease in *Mct1*^*+/−*^ (purple). **f** PSTH spike count: spike sum across ten trials in 1-ms time windows (teal). Latency: time when PSTH surpassed 1.5× baseline (pink). **g** IC response strength in *Mbp*^*shi/shi*^ mice was significantly increased (two-sided Wilcoxon rank-sum test, *P* = 0.05, *t* = 1.989). **h**–**j** Auditory cortex response strength was unchanged for all mouse mutants (*Mbp*^*shi/sh*^i, *Mbp*^*neo/neo*^, and *Mct1*^*+/−*^, respectively). **h** *Mbp*^*shi*^ mice (two-sided Wilcoxon rank-sum test, *P* = 0.72, *t* = 0.673; *n* = 9 mutants; *n* = 12 Wt). **i** Mbp^*neo*^ mice (two-sided Wilcoxon rank-sum test, *P* = 0.17, *t* = −2.06; *n* = 8 mutants, *n* = 8 Wt). **j** *Mct1* mice (two-sided Wilcoxon rank-sum test, *P* = 0.15, *t* = −2.803; *n* = 6 mutants; *n* = 8 Wt). **k**–**n** Response latency was increased in *Mbp*^*shi/shi*^ mice in both the **k** IC (two-sided Wilcoxon rank-sum test, *P* = 0.0012, *t* = 5.728, *n* = 6) and **l** ACx (two-sided Wilcoxon rank-sum test, *P* = 0.023, *t* = 4.887, *n* = 12) compared to Wt (*n* = 7 and *n* = 9, respectively). **m** *Mbp*^*neo/neo*^ mice (orange, *n* = 8) compared to Wt (black, *n* = 8, two-sided Wilcoxon rank-sum test, *P* = 0.0012, *t* = 6.424), and **n** borderline in *Mct1*^*+/−*^ mice (purple, *n* = 6; two-sided Wilcoxon rank-sum test, *P* = 0.06, *t* = 2.212). All bar plots: mean data for all animals and error bars the S.E.M. In **b**–**f**, S.E.M. is the shaded area, and individual data points are individual animals. Outliers are depicted with an x and were not considered in the statistical analysis. Source data are provided as a Source Data file.

While repeated responses to sounds with a temporal structure were clearly affected in the myelin mutants, single sound responses, measured through in vivo extracellular responses to single sound stimuli (i.e., clicks, Fig. 5a) in *Mbp*^*shi/shi*^ mice, were largely unaffected. Surprisingly, at the level of the IC, we obtained an increased response strength compared to Wt (Fig. 5b, g). However, in the ACx, response strength was similar to Wt both in *Mbp*^*shi/shi*^ (Fig. 5c, h) and *Mbp*^*neo/neo*^ mice (Fig. 5d, i). In contrast, response latencies (Fig. 5f) were increased in *Mbp*^*shi/shi*^ at both auditory stations (Fig. 5b, c, k, l) similar to *Mbp*^*neo/neo*^ mice (Fig. 5d, m), and this increase correlated with the degree of dysmyelination. Not surprisingly, in myelinated *Mct1*^*+/−*^ mice, cortical neurons had Wt-like responses to simple clicks in relation to latencies (Fig. 5e, n). Only the *Mct1*^*+/−*^ mice showed a tendency toward less responsiveness that did not reach significance (Fig. 5e, j). Response jitter (variability in the onset response; Supplementary Fig. S7A) and intertrial reliability (Supplementary Fig. S7F) were not different in any of the models or stations (Supplementary Fig. S7B–S7E, S7G–S7J).

### Spectral processing of pure tones

**Fig. 6:**
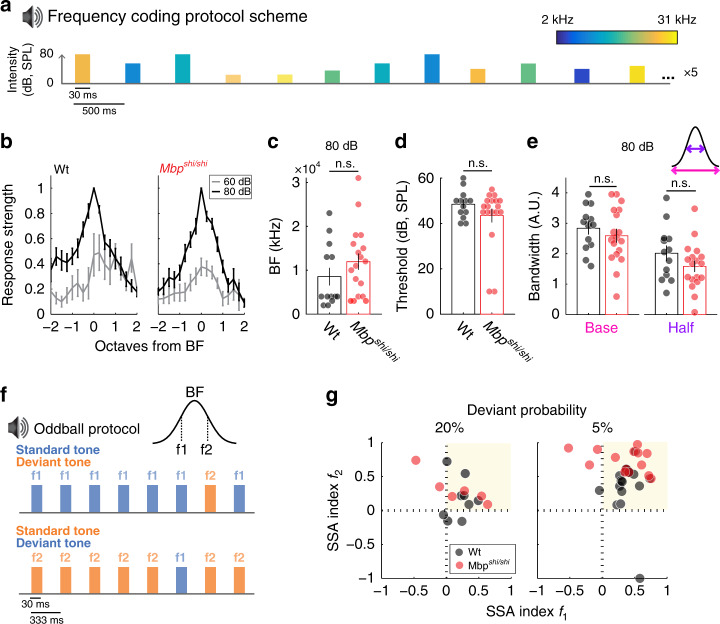
Frequency responses, tuning, and discrimination are not affected with dysmyelination in the ACx. **a** Schematic of the tone-sweep protocol used to test tuning. Twenty-four 30-ms-long, pure tones (2–31 kHz) were played at different intensities in ten repetitions (Rep.) of each frequency-intensity combination in random order. **b**–**e** Basic tuning properties are not affected with dysmyelination. Wt: *n* = 13 recordings, ten mice. *Mbp*^*shi/shi*^: *n* = 18 recordings, 15 mice. **b** Normalized tuning curves for Wt (left) and *Mbp*^*shi/shi*^ mice showing selectivity in octaves from best frequency (BF) at 60 (gray) and 80 dB (black). **c** Recordings from comparable rostrocaudal locations yielded comparable BFs at 80 dB (two-sided Wilcoxon rank-sum test, *P* = 0.25, *t* = 1.839) indicating normal tonotopy. **d** Auditory cortical thresholds were comparable between groups (two-sided Wilcoxon rank-sum test, *P* = 0.51, *t* = −1.638). **e** Tuning bandwidth was comparable between groups (two-sided Wilcoxon rank-sum test, *P* = 0.56, *t* = −1.148, and *P* = 0.21, *t* = −2.363 for base and half-bandwidth, respectively). **f** Schematic of the oddball protocol used to test stimulus-specific adaptation. Two tones differing in a Δ*f* of 10% were presented (rate 3 Hz) with different probability. The standard tone was presented with high probability (80 or 95% of trials) and the deviant tone with low probability (20 or 5%). **g** Stimulus-specific adaptation indices (normalized difference between deviant and standard response) for the two tones were plotted against each other. For low deviant probabilities (Dp = 5%), indices were above 0.3, indicating that deviant responses were at least twice as large as standard. This effect diminished as the probability of the deviant sound increased. While no difference in SSA was observed between the groups at Dp = 20% (one-way ANOVA, *P* = 0.35, Wt: black, *n* = 11 recordings; *Mbp*^*shi/shi*^: red, *n* = 14 recordings), for Dp = 5%, *Mbp*^*shi/shi*^ mice showed even more pronounced SSA (one-way ANOVA, *P* < 0.001; Wt: *n* = 9 recordings. *Mbp*^*shi/shi*^: *n* = 6 recordings). All plots represent the mean data of all recordings per group and error bars the S.E.M. and individual data points are individual animals, except for SSA, where they are recording sites. Source data are provided as a Source Data file.

Key features of spectral processing are frequency tuning and response adaptation, both of which are strongly influenced by neuronal integration, i.e., the functional convergence of stimuli onto individual cortical neurons. Frequency tuning, a property of the auditory system, builds on frequency-specific inputs onto a given cell, and is modulated by lateral inhibition. Thus, differential conduction velocities in converging pathways as a result of reduced myelination could have a lasting effect on neuronal integration. Surprisingly, upon stimulation with pure tones (Fig. 6a), tuning curves had comparable shapes in *Mbp*^*shi/shi*^ and Wt mice, as a function of sound intensity (Fig. 6b), and covered comparable best-frequency (BF) ranges within the sampled regions (Fig. 6c). There was no difference between groups in hearing thresholds (Fig. 6d) or tuning bandwidth (Fig. 6e), which depends largely on convergent inputs^66^.

Adaptation is crucial for sensory filtering in the auditory system^67,68^ and a measure of neuronal integration. It is typically tested using oddball paradigms (Fig. 6f), in which two frequencies that elicit responses of similar magnitude are presented sequentially such that one appears with higher probability (standard) than the other (deviant). Here, stimulus-specific adaptation (SSA) is reflected in a decreased response to the standard tone, while the response to the deviant tone remains constant or increases^68,69^. Importantly, cortical SSA was not reduced in *Mbp*^*shi/shi*^ compared to Wt mice (Fig. 6g). Thus, widespread dysmyelination had no obvious effect on the integration of auditory stimuli, presumably because the convergent inputs were all similarly delayed.

**Fig. 7:**
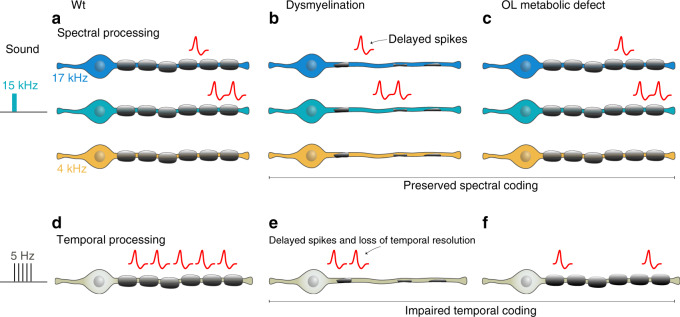
Role of oligodendrocytes in information processing extends beyond conduction velocity regulation to energy support of axons and axonal excitability regulation. **a** Schematic illustrating parallel processing of pure tones in a Wt animal. Sound presentation (left, teal, 15 kHz) activates fibers sensitive to 15 kHz (middle, teal) more strongly than fibers sensitive to 17 kHz (upper, blue, 17 kHz), and does not activate fiber sensitive to 4 kHz (lower, yellow, 4 kHz). **b** With dysmyelination, spectral processing is unaffected, but delayed responses are observed. **c** Oligodendrocyte metabolic defects affect neither the latency nor strength of responses to simple tones. **d** Temporal processing of continuous stimuli (i.e., presentation of clicks at 5 Hz) in a Wt animal. **e** Temporal processing is affected with dysmyelination beyond the increase in conduction velocity (delayed spikes). We observe loss of temporal resolution in both dysmyelination conditions and **f** with loss of oligodendrocyte metabolic stability.

Finally, in Fig. 7, we provide a summary of the auditory abnormalities observed in our mouse models in which parallel processing of pure tones is not affected by dysmyelination, but in-line temporal processing is affected by myelin disturbances related to either loss of myelin per se or a reduction in the glial metabolic support function.

## Discussion

To explore the role of myelinating oligodendrocytes in information processing, we studied the detection and perception of auditory stimuli in mice. We used auditory brainstem potentials and multiunit recordings in the auditory cortex of mutant mice, as well as behavioral readouts in awake animals, to compare the effect of dysmyelination in partially (*Mbp*^*neo/neo*^) and complete (*Mbp*^*shi/shi*^) MBP-deficient mice with that of an isolated reduction of axoglial metabolic support (*Mct1*^*+/−*^). The combined results reveal that dysmyelination impairs sustained stimulus detection, a relevant feature also of human speech processing. Interestingly, the deficits in temporal processing upon dysmyelination were partially comparable to those resulting from a reduction of axoglial metabolic support, even in the absence of dysmyelination. The myelin sheath increases conduction velocity^2^, but it may be the tight axonal contact that facilitates metabolic support^1^. Dysmyelination results in conduction delays (measured through ABRs and cortical evoked responses) and desynchronization of inputs (measured through ABRs). However, lack of myelin also results in a maldistribution of axonal channel proteins at the node^51,70–73^, whose functional effect we have confirmed with potassium channel blockers that partially attenuated the effect of dysmyelination on optic nerve action potentials. The metabolic mutant, however, is not dysmyelinated and unlikely to suffer from any channel misdistributions. Yet, these mutant mice also showed a deficit in temporal reliability and acuity, partially similar to the observation in shiverer mice. This suggests that axoglial metabolic support is necessary for temporal auditory processing at some level. Taken together, the data indicate that myelinating oligodendrocytes play an important role in sensory processing and perception.

The subcortical delays in the dysmyelinated mice were accompanied by reduced synchrony of auditory brainstem potentials, a feature not observed in mutants with merely reduced glial metabolic support. The decreased amplitudes and shapes of waves II and III, most likely reflect a desynchronization of converging inputs at the level of the cochlear nucleus and olivary complex (where ascending auditory signals become bilateral). The later waves IV and V showed instead an increase in amplitude in dysmyelinated mutants, most likely reflecting a compensatory mechanism to the loss of synchrony at the earlier stations. Similar gain increases have been reported in mice deprived of auditory stimuli^74^, as a result of aging^44^ or in humans with multiple sclerosis lesions^75^.

While dysmyelination had a strong effect on conduction velocity, spectral processing of simple stimuli (pure tones) in the primary auditory cortex appeared remarkably unaffected. Auditory information can reach the IC and then the ACx through different parallel pathways that might be differentially affected by dysmyelination. While simple click sounds and pure tones are likely activating a limited (possibly the most direct) subset of sound-encoding circuits, responses to these stimuli were unaltered, indicating that basic relay processing and sound integration was not affected by the absence of myelin. However, we detected deficits in temporal processing unexplained by reduced conduction velocity alone. Temporal reliability and acuity were affected in all mutants. Unexpectedly, the magnitude of this defect was correlated with the degree of hypomyelination, but was not limited to dysmyelination phenotypes. It was also a feature of *Mct1*^*+/−*^ mice, in which myelinated axons suffer from reduced glial metabolic support^4^. While reduced *Mct1* expression affects presumably both oligodendrocytes and astrocytes, these heterozygous mice exhibit defects of the myelin architecture with aging support^4^.

Behavioral tests in dysmyelinated mutants (*Mbp*^*neo/neo*^) demonstrated that the temporal processing deficits, as detected by cortical multiunit recordings, were paralleled at the perceptual level. Myelinating oligodendrocytes are thus involved in the temporal processing of auditory signals, which has previously been implicated in higher CNS functions in humans, such as speech recognition^76–78^. Here, the phenotypic similarities of the two types of glial defects (i.e., dysmyelination in *Mbp* mutants and isolated metabolic defects in the *Mct1*^*+/−*^) raise the possibility that a compromised metabolic support of axon function is the common denominator of all these auditory phenotypes. Our data cannot rule out the possibility that reduced conduction velocity by itself has a similar phenotypical effect, which remains unproven as the lack of myelin is invariably linked to elevated axonal energy consumptions. We note, however, that regardless of the mechanisms, the oligodendrocyte defects affect several auditory functions at the network level and in the absence of any signs of axonal degeneration.

For example, myelination defects affected the coding of a stimulus’ temporal envelope, reflected as fatigability, i.e., a lack of temporal reliability. For *Mbp* mutants, it was unexpected that the responses to longer trains of clicks diminished or even stopped after five to six repetitions of the stimulus, especially when the intervals between consecutive clicks were relatively large (200 ms, repetition rate 5 Hz).

Myelin speeds axonal conduction velocity and reduces energy consumption by restricting action potentials that are metabolically expensive^79^ to the nodes of Ranvier^2,19,80^. Evidence for this latter role is accumulating. For instance, the high density of sodium channels necessary for repetitive firing^81^ is energetically not feasible in the absence of myelin. While axon caliber correlates with myelin thickness^82^, this is not the general rule^83–85^. Myelination alone is, in fact, not a good predictor of conduction speed^19^, and its thickness has been suggested to better correlate with axonal firing frequency, in other words, with the energetic needs^86^.

Here, the altered distribution of sodium and potassium channels in unmyelinated axons is critical. In *Mbp*^*shi/shi*^ mice, sodium channels are increased^70,87^, and the developmentally high expression of Na_v_1.2 does not switch to that of Na_v_1.6 channels^72,88^. This might contribute to increased fatigability in *Mbp*^*shi/shi*^ mice since Na_v_1.6 channels are associated with—and necessary for—repetitive firing^89^, in the absence of associated Na_v_β4 subunits^90^. In addition, an elongation of nodes^51,91^ and overexpression of K_v_1.1 and K_v_1.2 channels has been reported in shiverer mice^51,73^. K_v_1 channels are normally clustered at juxtaparanodal domain^92^, but in dysmyelinated axons abnormally exposed to the extracellular space causing increased K_v_1-mediated potassium efflux. Moreover, in the absence of myelin, oligodendrocyte-dependent potassium siphoning would be reduced^93–95^. Together, this might lead to a greater activity-driven accumulation of extracellular potassium, resulting in a longer time course of membrane hyperpolarization. Interestingly, the reduction in the lengths of the AIS in *Mbp*^*shi/shi*^ mice may explain the absence of hyperexcitable responses in ACx compared to IC, as a short AIS is typically associated with reduced intrinsic neuronal excitability^53,96^.

In agreement with this hypothesis, when potassium channels in shiverer mice were acutely blocked with 4-AP before ex vivo electrophysiological recordings of optic nerves (our experimentally accessible model of a dysmyelinated tract), we noted a partial restoration of CAP amplitude and a smaller hyperpolarization phase. A distinct effect of dysmyelination, and associated with increased potassium fluxes, is the enhanced energy consumption by axonal Na^+^/K^+^ ATPases, which contribute to the axon’s repolarization and sustained ability to fire at high frequencies^97^. All these data are consistent with the efficacy of 4-AP as a symptomatic treatment of patients with MS^98^.

In addition to the loss of temporal reliability, we determined a myelination-dependent defect of temporal acuity, i.e., a failure in the coding of rapid changes in the temporal structure of the stimulus. Here, a powerful paradigm is sound gap detection^47^, a function that relies on cortical auditory processing^57,99,100^. In humans, poor performance in gap-detection tests is closely associated with a corresponding loss of accurate speech discrimination^101,102^. We found that cortical neurons of dysmyelinated shiverer mice failed to detect silent gaps within a stream of white noise when these gaps were shorter than 3 ms, a marked deviation from control mice that detect gaps as short as 0.5–1 ms. Hypomyelinated *Mbp*^*neo/neo*^ mice exhibited a milder deficit.

Poor gap detection in *Mbp*^*shi/shi*^ mice could be caused by longer refractory periods and reduced excitability of the axons secondary to the abnormal distribution of ion channels. In the *Mbp*^*neo/neo*^ mice the deficit is milder. Thinner myelin in these mice could result in stronger depolarization of the internodal membrane^2^, and delayed repolarization that might explain the decreased conduction velocities and poor gap detection. In the metabolic mutants, on the other hand, there is no evidence of myelin structural changes or conduction delays, and thus the gap-detection deficits can be attributed to the diminished energy supply to the axon. These data suggest that reduction in the oligodendrocyte energetic support to the axon alone is sufficient to cause poor gap detection. An in-depth analysis of the location and individual electrophysiological properties of cortical Na^+^ and K^+^ axonal channels is required to further understand their role during repetitive firing in our mutant models. Future research will help us better understand the extent to which the synaptic connectivity of inhibitory and excitatory neurons in cortical circuits depends on metabolic supply.

All multiunit recordings were performed in anesthetized mice. We note that key aspects of auditory processing occur pre-attentively and should be unaffected by anesthetics^103^. However, neuronal circuits with inhibitory input can behave differently under anesthesia, which can bias experimental results. Thus, it was important to parallel the electrophysiological results with behavioral experiments on auditory perception in freely moving animals. Since motor-impaired *Mbp*^*shi/shi*^ mice could not be used for these experiments, we analyzed mice homozygous for the newly created *Mbp*^*neo/neo*^ allele, which had no visible motor defects and was long-lived. In this mutant, we confirmed poor gap detection at the perceptual level by operant conditioning of auditory stimuli and responses. The behavioral test was in fact more sensitive than the cortical recordings, in that the deficit extended over even longer gaps. This is not surprising given that the behavioral response depends on the integration of information about the gap and the startle sound, which includes, in addition to cortical responses, the circuits that regulate behavioral inhibition. We observed no such auditory deficits in heterozygous shiverer mice, which exhibit (unlike *Mbp*^*neo/neo*^ mice) only a very minor hypomyelination^36^. This suggests that a threshold level of hypomyelination is required for the auditory phenotype.

The aim of our study was to use different mouse mutants with structural myelin defects and compromised metabolic support of axons to explore the role of myelination at the network level. While the chosen genetic defects preferentially affect myelin structure or metabolic support, the desired effects cannot be completely uncoupled from each other. Hypomyelination will inevitably affect both the fine structure and the energy balance of an axon, whereas metabolic defects of glial cells will have secondary effects, such as neuroinflammation, that can feed back on myelin structural integrity. Nevertheless, this mixture of abnormalities is distinct for each animal model and demonstrates that the integrity of the axon–myelin unit is important for network functions in the cortex. Here, we show that dysmyelination impairs sensory perception and demonstrates physiological abnormalities at different levels of the auditory pathway. Moreover, we could mechanistically distinguish between reliability defects and acuity deficits of auditory processing. These studies, which included experiments in freely moving mice, revealed a role of myelin-forming oligodendrocytes in information processing that might go beyond the speeding of neuronal responses. We discovered that myelin influences sustained and precise axonal firing, essential to properly code auditory stimuli. Moreover, specific phenotypic similarities between mutants with a predominant dysmyelination versus predominant metabolic defect strongly suggest that energetic failures of myelinated axons can be a mechanism of auditory dysfunctions. Auditory phenotyping emerges as a promising experimental system to investigate the role of oligodendrocytes and myelination in information processing.

## Methods

### Mice

All mice were housed in standard plastic cages with 1–5 littermates in a 12-h/12-h light/dark cycle (5:30 am/5:30 pm) in a temperature-controlled room (~21 °C), with ad libitum access to food and water. All mice used were bred under the C57BL6/N background. Data obtained from male and female mice were pooled together, unless otherwise stated. The experimental and surgical procedures were approved and performed in accordance with the Niedersächsisches Landesamt für Verbraucherschutz und Lebensmittelsicherheit (license numbers 33.19-42502-04-16/2337 and 33.19-42502-04-14/1465).

Homozygous shiverer mice were obtained by crossing heterozygotes (*Mbp*^*shi/+*^). In all experiments, *Mbp*^*shi/shi*^ mice and their control littermates (Wt) were 6–12 weeks of age, unless otherwise stated. In accordance with the Verhaltensversuche zur Phänotypisierung von Mausmodellen neurodegenerativer und neuropsychiatrischer Erkrankungen (license number 33.9-42502-04-10/0288), a new hypomyelinated mouse with reduced *Mbp* expression (<50%), was generated by homologous recombination in ES cells, using a modified *Mbp* gene carrying a LacZ-neomycin cassette upstream of exon 1. Successful targeting disrupted the 5′ regulatory region without affecting the larger GOLLI transcription unit (Supplementary Fig. S1A). Homozygous *Mbp*^*neo/neo*^ mice were born at the expected frequency. For all experiments, *Mbp*^*neo/neo*^ and controls (Wt) were used at age 9.5–14 weeks, unless otherwise stated. *Mct1*^*+/−*^ mice^4^ were kindly provided by Pierre Magistretti (Lausanne), and heterozygous and control littermates were analyzed at the age of 10–14 weeks. Primers used for genotyping can be found in the Supplementary list of primers.

### Electron microscopy

After transcardial perfusion fixation (4% formaldehyde and 2.5% glutaraldehyde in phosphate buffer, pH 7.3)^104^, for the ACx, mouse brains, 8–12 weeks of age, were dissected, and sagittal or coronal vibratome (Leica VT1000S) slices of 200–300-µm thickness were prepared. A punch of the region of interest was taken, prepared for electron microscopy^105^, and embedded in Epon. Ultrathin sections were prepared using a Leica Ultracut S ultramicrotome (Leica, Vienna, Austria) and imaged with a LEO912 electron microscope (Zeiss, Oberkochen, Germany) using a 2k on-axis CCD camera (TRS, Moorenweis, Germany).

### Auditory brainstem responses

Auditory brainstem responses (ABRs) were measured as described^106^. Mice were anesthetized with an intraperitoneal injection of 250 mg/kg Avertin (mixture of 2,2,2-tribromoethyl alcohol 2%, Sigma-Aldrich, and tert-amyl-alcohol 2%, Merck), except for the Mbp^*neo/neo*^ line, which was treated as in ref. ^107^ and anesthetized with ketamine (125 mg/kg) and xylazine (2.5 mg/kg) i.p. Temperature was kept at 36 °C via a heating pad (World Precision Instruments, ATC 1000). Subdermal needles (BD Microlance, 30 G ½”, 0.3 × 13 mm) were placed at the vertex (active electrode), the left pinna (reference), and the back of the animal (active shielding). Click stimuli were ipsilaterally delivered from a speaker located ~9 cm from the left ear. Square click waveforms (0.03-ms long) were presented at 20 Hz (50-ms intertrial intervals (ITI)) at different intensities (0–80 dB). The difference in potentials was amplified 10,000 times using a custom-made amplifier. A National Instruments shielded I/O connector block (NI SCB-68), interfaced in a Matlab environment, was used for data acquisition (50,000-Hz sampling rate). Recorded voltage traces were bandpass-filtered offline (300–3000 Hz) using a Butterworth filter. Data were cut from the stimulus-presentation onset (0 ms) in 12-ms windows. Trials with heart rate artifacts (wave shapes larger than ±4.7–9.2 μV) were removed, and 1000 trial repetitions of each stimulus were used for data analysis.

### Acoustic stimulation

All sound stimuli were digitally synthesized and presented using Matlab (The Mathworks^®^, USA), at a sampling rate of 98 kHz and in a pseudorandom order. There were typically ten repetitions of each stimulus, unless otherwise stated. The intensities were measured in decibel sound-pressure levels (dB-SPL). Sounds used for electrophysiology were delivered by a USB audio interface (Octa-capture, Roland, USA), amplified with a Portable Ultrasonic Power Amplifier (Avisoft, Germany) and played in a free-field ultrasonic speaker (Ultrasonic Dynamic Speaker Vifa, Avisoft, Germay). Calibration of the testing apparatus was made using BBN and click sounds at different intensities with a Brüel & Kjaer (4939 ¼”) free-field microphone, with Brüel & Kjaer amplifier (D4039, 2610, Denmark). All experiments were performed in a sound-attenuated and anechoic room.

### Acute electrophysiology

Prior to surgery, mice were anesthetized and maintained as reported for the ABR procedure. Mice were placed on a stereotaxic apparatus using inverted ear bars to avoid damage to the ear canal (World Precision Instruments, Sarota, FL USA, 502063). A cut was made along the midline and the skull exposed and cleaned of adherent tissue with a scalpel and hydrogen peroxide. A metal screw (M1×1, Germany) was inserted into the right parietal cortex and used as ground. A metal post was glued on the skull frontal to lambda with dental cement (Unifast, TRAD), this allowed for removal of the ear bars. The muscle temporalis was detached from the skull and a 4 × 2-mm craniotomy was performed using a dental drill (World Precision Instruments, Omnidrill3, tip #7), following the contour- delimited rostral and ventrally by the squamosal suture, dorsally by the temporal ridge, and caudally by the lambdoid suture. The speaker was placed ~13 cm from the right ear of the mouse. At the end of every experiment, mice were euthanized by anesthesia overdose. The brain was removed, and immersion fixed in 4% PFA. After 24 h, the brain was washed with PBS 1× and stored in 30% sucrose.

Recordings were performed with glass-covered tungsten electrodes (AlphaOmega, Germany) or platinum/tungsten glass-coated electrodes (Thomas Recordings, Germany) with impedances between 1.5 and 2 MΩ. In 30% of the recordings, the electrode was stained with Dye I (dioctadecyl-tetramethylindocarbocyanine perchlorate, Aldrich, 468495) dissolved in absolute ethanol, before insertion, for later visualization of its position. After the craniotomy, a drop of saline was used to clean the surface of the brain. The electrode was inserted perpendicular to the surface of the primary auditory cortex^108^ using a micromanipulator (Kopf, Inc., Germany). We recorded extracellular multiunit (MUA) sound-evoked responses in layer 3/4 of the ACx of anesthetized mice. Responses were characterized by onset latency and shape. ACx recordings in *Mbp*^*shi/shi*^ mice were done at a depth of ~405 μm (62-μm standard deviation (sd)) without differences in depth between control and mutant mice (*P* = 0.71). In the *Mbp*^*neo/neo*^ line, depth of recording was on average ~350 μm (60.5-μm sd) without differences between groups (*P* = 0.52). In *Mct1*^*+/−*^ mice, recordings were on average at ~380-μm depth (37-μm sd) (*P* = 0.32).

Electrophysiological signals were acquired at 32-kHz sampling rate, preamplified (HS-36-Led, Neuralynx, USA), and sent to an acquisition board (Digital Lynx 4SX, Neuralynx, USA) to yield the raw signals, which were acquired using a bandpass filter (0.1 or 200–9000 Hz) and stored for offline analysis. Recording and visualization of the data was made using the Cheetah Data Acquisition System software (Neuralynx, USA). For multiunit analysis (MUA), spikes that are not attributed to a single neuron, the signals were high-pass filtered at 350 Hz. For spike detection, a threshold of 6 times the mean absolute deviation from the median of the filtered voltage traces was used. For the analysis, only recordings that had significant auditory-evoked responses at any sound intensity compared to a 200-ms presound baseline activity (paired t test) were used.

A click-rate protocol (Fig. 2a) was used to assess temporal reliability. Each was a 0.05-millisecond long positive step function. Bursts of ten clicks were presented every 6 s, each at a different rate (2–50 Hz). The first click of each burst was used to characterize latency, amplitude, reliability, and jitter of auditory response (Fig. 5 and Supplementary Fig. S7). For the latency measurement, we only included the spikes that occurred at least 10 ms after sound onset, given latencies reported for the ACx in anesthetized mice^108^. To measure reliability of repeated responses (Fig. 2d, e), synchronicity measurements were obtained from a measure of the vector strength^109^ of the spikes occurring for the duration of the stimulation in all repetitions of the stimulus, which were obtained by converting each spike time (*τ*_i_) as a circular vector with phase (*θ*_i_) between 0 and 2π, according to the stimulus phase of a specific rate. All the spikes occurring after each of the ten clicks were selected in terms of their latency with respect to the previous click. A window equivalent to the phase duration (equal to the interclick interval) was selected for each click-rate presentation. The spike phases were then expressed in terms of

1 $$\theta _{\mathrm{i}} = 2{\uppi}\ast ({\mathrm{mod}}(\tau _{\mathrm{{i}}}{\rho}))/{\rho},$$

here, *τ*_i_ is the latency of each spike, *θ*_i_ is the phase representation of each spike, and *ρ* is the phase of the stimulus presented (e.g., for a click train at 5 Hz, *ρ* = 0.2). To calculate the percentage of spike synchronicity, the total spike-phase distribution was binned in 20 bins from 0 to 2π, and the spike count of the bin with the maximum synchronization in the first half of the vector (π), was taken. This spike count was expressed in terms of percentage, which means the percentage of spikes that were fully synchronized per stimulus in one recording.

A gap-in-noise detection paradigm was used to evaluate temporal acuity. Once every second, 200-ms-long broadband noise (BBN, pregap) sound was followed, after a short silent gap, by a 50-ms- long BBN sound (post gap). Silent gaps durations were 0, 0.5, 1, 2, 3, 4, 5, 7, 10, 20, 50, and 100 ms (Fig. 3a). Rise/fall time of the BBN pulse was 1 ms. For gap-detection analysis, only recordings with a significant response to the mean of the pregap sound (sum of spikes over a 100-ms window after stimulus onset), compared to presound baseline, were used (paired *t* test). For each animal, recordings at different locations were averaged. Additionally, only those animals that had a significant response to the post-gap BBN following a 100-ms gap were included in the analysis (100-ms window from the start of the post-gap BBN). Peristimulus time histograms (PSTH) were generated by adding spikes across trials in 1-ms bins over a 100-ms window (Fig. 3d). The pregap response (PSTH in a 100-ms window) was generated from all gap trials, since the pregap response should not be affected by the gap that follows it. For the post-gap responses (PSTH over 100 ms), each gap was treated separately. Comparisons between control and *Mbp*^*shi/shi*^ and *Mbp*^*neo/neo*^ mice revealed no significant differences in the amplitude of the pregap response (ANOVA, *P* = 0.53 and *P* = 0.6, respectively). Also, the peak of the post-gap response was used for group comparisons. Since some groups showed increased latencies, the PSTHs were centered at their peaks. An ANOVA was performed in a 21-ms window centered on the peak.

For the analysis of the gap detection versus baseline, a 50-ms window of baseline activity was compared to a 50-ms window of the post-gap response for each recording site using a paired *t* test.

Response latency was measured as the time at which the PSTH that surpassed 1.5 times the baseline activity amplitude was measured as the sum of the spiking activity across 10 repetitions of the first click stimuli of the 5-, 8-, 10-, 14-, and 20-Hz condition. To measure tonotopy, pure tones of variable frequencies and intensities were presented (24 tones with frequencies between 2 and 31 kHz, and intensities between 0 and 80 dB) (Fig. 6a). Each tone was 30-ms long, had on/off ramps of 5 ms. Presentation rate was 2 Hz (500-ms intertrial interval). Five repetitions of each frequency/intensity combination were presented in a random order. The analysis was performed on individual recording sites, although in many mice, recordings were obtained from several sites along the ACx.

For the reconstruction of the single-site tuning (Fig. 6b), the spikes elicited by each frequency in a 200-ms window after stimulus onset were added. The tuning curves (TC) were smoothened using a zero-phase digital hamming filter with a window of four points. The smoothened TC was then used to calculate the best frequency (BF, the frequency that elicited the maximum number of spikes at 60 and 80 dB). In normalized tuning curves, activity was expressed as a function not of frequency but of the distance (in octaves) from the best frequency. Response thresholds were obtained by visual inspection (blinded as to the genotype) of a 200-ms window from stimulus onset. Tuning curve width, both at the base and the half-distance from the peak activity (Fig. 6e), was calculated as the *z* score of the sum of spikes over a 60-ms window from stimulus onset at all frequencies and intensities.

Frequency oddball paradigms were evaluated presenting two pure tones (*f*_1_ and *f*_2_) separated in frequency by Δ*f* = 0.1 as shown by

2 $$\Delta f = (f_2-f_1)/(f_2 \times f_1)^{1/2},$$

*f*_1_ and *f*_2_ were centered on the best frequency of the MUA at each recording site. Three different probabilities of presentation of the deviant sound were used (5%, 10%, and 20% of appearance, at 3 Hz). Each combination of stimuli (Δ*f*, % of appearance and rate) was presented 300–500 times.

The SSA index (SSAi) was calculated as described in ref. ^110^

3 $${\mathrm{SSAi}}\left( f{_{\mathrm{i}}} \right) = ({\mathrm{d}}\left( {f_{\mathrm{i}}} \right) - {\mathrm{s}}\left( {f_{\mathrm{i}}} \right))/\left( {{\mathrm{d}}\left( {f_{\mathrm{i}}} \right) + {\mathrm{s}}\left( {f_{\mathrm{i}}} \right)} \right),$$

where _*i*_ = 1 or 2 and d(*f*_i_) and s(*f*_i_) are responses (as normalized spike counts) to frequency *f*_i_ when it was deviant or standard, respectively. With increasing Δ*f* (e.g., 20%), we observe a greater SSAi, since the response to the deviant is larger due to the greater difference between *f*_1_ and *f*_2_. This happens because similar frequencies are generalized and are susceptible to adaptation. A small Δ*f* (e.g., 5%) will generate a reduced SI. Changing the presentation rate also modifies the SSAi, since very low rates of presentation (e.g., 1 Hz) will not generate a continuity of the sound, and the sounds will be computed as random events. The higher the presentation rate (e.g., 3 Hz), the easier to detect deviant stimuli and the larger the SI. In addition, the probability of deviant presentation also modifies SSAi. If a deviant sound is very rare (e.g., dP = 5%), its detection will be easier than if the sound occurs more frequently (e.g., dP = 20%).

### Gap prepulse inhibition of the acoustic startle reflex

We used behavioral Gap-PPI to test behavioral temporal acuity^59^. This test was performed in *Mbp*^*neo/neo*^, *Mbp*^*shi/+*^, and their control littermates. We used 9 control animals (Wt pulled together from both dysmyelination lines), 6 *Mbp*^*neo/neo*^, 6 *Mbp*^*shi/+*^. All mice were between 9 and 16 weeks at the age of testing. For testing, a mouse was confined in a custom-made Plexiglas tunnel (12-cm long by 4-cm diameter), placed above a piezo element (TRU components, 800-ohms impedance, 50-mm diameter, spanning 30 V) (Supplementary Fig. S6A). The piezo was connected to a data acquisition system (NI SCB-68). Data acquisition (1-kHz sampling rate) was controlled with a custom-made code in Matlab and sound delivery was done using presynthetized sound tracks in Matlab, played through VLC media player (Video Lan Organization).

The testing protocol was as summarized in Supplementary Fig. S6B. Mice were initially acclimatized to the setup for 10 min before the start of the experiment: 5 min of silence followed by 5 min of exposure to the background sound (BBN of 70 dB). Testing was then initiated. The startle noise was a BBN 105 dB with 40-ms duration. It appeared every 10–20 s at random times to avoid startle prediction. The startle could be preceded by a gap (0-, 1-, 2-, 3-, 5-, 7-, 10-, 25-, or 50-ms long), which ended 50 ms before the startle (Fig. 4a). Gaps and startle had on/off ramps of 1 ms. The session started with 10 startle-only pulses, followed by gap-preceded startle presentations (ten startle presentations per gap length). The experiment ended with five startle-only pulses to assess habituation. The complete presentation of 105 trials had a duration of ~30 min.

Gap-PPI analysis was done as described before^59^, with mild modifications. The magnitude of the acoustic startle reflex (ASR) was measured as the maximal vertical force (peak-to-peak voltage output) exerted on the piezo element in a 500-ms window starting with the onset of the startle noise. Baseline activity (2 times the root mean square of the voltage trace in a 500-ms window before the startle noise) was subtracted. Startle and baseline amplitude were measured in arbitrary voltage units provided by the piezo element. Noisy trials (three times the standard deviation of the root mean square of a 500-ms window before gap presentation) were discarded from the analysis. The percentage of prepulse inhibition for each gap and mouse was calculated as the following:

4 $${\mathrm{PPI}}\left( \% \right) = 100^\ast \left( {{\mathrm{ASR}} - {\mathrm{ASRx}}} \right)/{\mathrm{ASR}},$$

where ASR is the startle response elicited at 0-ms gap, and ASRx is the startle response elicited per gap played. The data were fitted with a generalized logistic function:

5 $${{f}} = - a/2 + \left( {a/1 + {\mathrm{exp}}(b + c * {{x}})} \right.$$

Recordings with a fit coefficient (*R*^2^) below 0.6 were excluded from the analysis.

The gap-detection threshold was considered as the value of the fitted curve that elicited 50% of the maximal inhibition achieved per mouse. A second normalization was then made to the longest gap (50 ms) presumed to elicit the maximum value of inhibition (100%). For the statistical comparisons of the gap-detection threshold and amplitude of startle, parametric or nonparametric *t* test was used. A two-way ANOVA was performed for the comparison of the inhibition of the ASR along the different gap lengths.

### Analysis generalities

Analyses and figure outputs were always performed in a Matlab environment. All confidence intervals correspond to *α* = 0.05. Significance corresponds to ^n.s.^*P* > 0.05, *P ≤ 0.05, **P ≤ 0.01, ***P ≤ 0.001, ****P ≤ 0.0001. Individual data points represent either individual animals or recoding sites, stated in every figure legend. Statistical analysis was parametric or nonparametric according to the Shapiro–Wilcoxon normality test. Paired comparisons were done using *t* test, or rank-sum and post hoc multiple comparisons were performed using Bonferroni correction after two-way ANOVA or Kruskal–Wallis test.

### Statistics and reproducibility

EM pictures are examples of reproducible phenotypes with at least *n* = 3 independent animals per group. Histology pictures are examples of reproducible phenotypes with at least *n* = 4 animals per group. Acute electrophysiology experiments were typically performed in cohorts of Wt and mutant littermates over several weeks. *Mbp*^*shi*^ and *Mbp*^*neo*^ mice were recorded in different years with reproducible effects. All *Mct1* mice were recorded over the span of a month. For ABRs and PPI experiments, large cohorts of Wt and mutant littermates were recorded over 1–2 weeks. Three replicates of the behavioral experiments in the AudioBox were made over 2 years.

### Reporting summary

Further information on research design is available in the Nature Research Reporting Summary linked to this article.

## Supplementary information

Supplementary information

Reporting Summary

## Data Availability

The raw data that support the findings of this study are available from the corresponding author upon reasonable request. Source data are provided with this paper.

## Source data

Source Data

## Code availability

No custom mathematical algorithm was used that is necessary for the analysis of the aforementioned data.
