# Supplementary material for: A role of oligodendrocytes in information processing
Source: Nat Commun. 2020 Oct 30;11:5497. doi: 10.1038/s41467-020-19152-7 (PMC7599337; doi:10.1038/s41467-020-19152-7)
Supplement: Supplementary file 1 — Supplementary information [file 41467_2020_19152_MOESM1_ESM.pdf]

Supplementary Information

Supplementary Figures

Figure S1

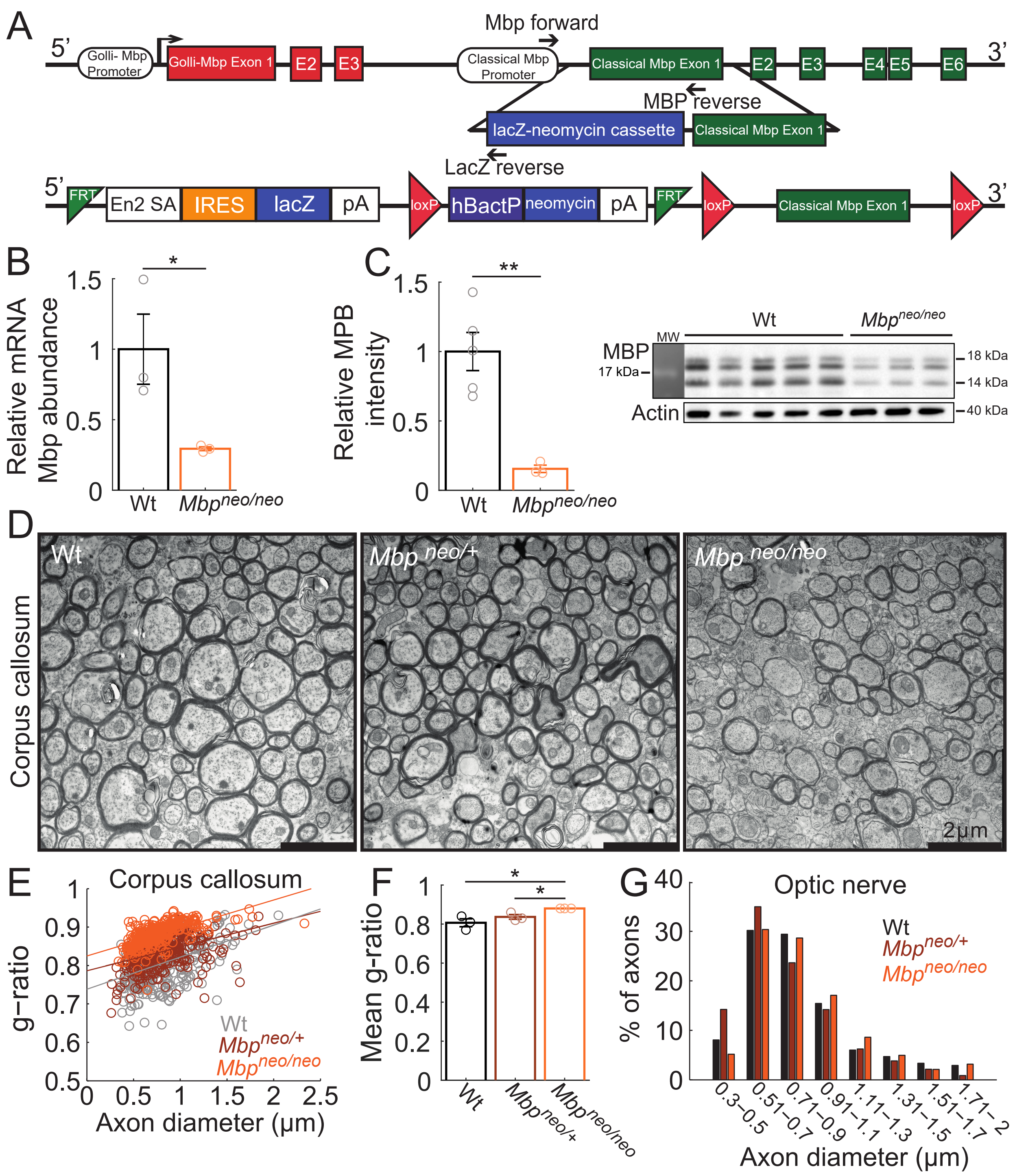
A
5'
Golli- Mbp Promoter
Golli-Mbp Exon 1
E2
E3
Mbp forward
Classical Mbp Promoter
Classical Mbp Exon 1
E2
E3
E4
E5
E6
3'
MBP reverse
lacZ-neomycin cassette
Classical Mbp Exon 1
LacZ reverse
5'
FRT
En2 SA
IRES
lacZ
pA
loxP
hBactP
neomycin
pA
FRT
loxP
Classical Mbp Exon 1
loxP
3'
B
Relative mRNA Mbp abundance
Wt
Mbp neo/neo
C
Relative MPB intensity
Wt
Mbp neo/neo
MW
Wt
Mbp neo/neo
MBP
17 kDa
18 kDa
14 kDa
Actin
40 kDa
D
Corpus callosum
Wt
Mbp neo/+
Mbp neo/neo
2µm
E
Corpus callosum
g−ratio
Wt
Mbp neo/+
Mbp neo/neo
Axon diameter (µm)
F
Mean g-ratio
Wt
Mbp neo/+
Mbp neo/neo
G
Optic nerve
% of axons
Wt
Mbp neo/+
Mbp neo/neo
0.3−0.5
0.51−0.7
0.71−0.9
0.91−1.1
1.11−1.3
1.31−1.5
1.51−1.7
1.71−2
Axon diameter (µm)

Figure S1. Molecular characterization of *Mbp*^{neo} model.

A) Genetic construct including the lacZ-neo cassette as provided by the European Conditional Mouse Mutagenesis Program (Eucomm). Eucomm consortium website with official depiction of construct: http://www.mousephenotype.org/about-ikmc/eucomm-program/eucomm-targeting-strategies. **B)** *Mbp* mRNA abundance is significantly decreased in *Mbo*$^{neo/neo}$ (orange, n=3 mice) compared to Wt (black, n=3 mice) (~70%, two-sided t-test, p=0.047, t=-53.05) **C)** MBP expression at the protein level showed a significant reduction in *Mbo*$^{neo/neo}$ (orange, n=3 mice) compared to Wt (black, n=5 mice) (80%, two-tailed t-test, p=0.0037, t=-31.87 for MBP). Right inset shows Western Blot used for quantification. Each column represents independent animals used for the quantification. **D)** Electron microscopy images of Wt (left) *Mbp*$^{neo/+}$ (middle) and *Mbp*$^{neo/neo}$ (right) caudal corpus callosum transverse sections. *Mbp*$^{neo/neo}$ mice have thinner compacted myelin than Wt. Scale bars: 2 µm. These results were reproducible between 3 biological replicates for each genotype. **E)** Distribution of g-ratios (axon diameter/axon + myelin diameter) per axon caliber in Wt (gray), *Mbp*$^{neo/+}$ (brown) and *Mbp*$^{neo/neo}$ (orange) caudal corpus callosum. **F)** G-ratio quantification revealed that *Mbp*$^{neo/neo}$ mice (orange) have significantly thinner myelin than Wt (black; two-tailed t-test, p=0.022, t=inf) and their heterozygote littermates *Mbp*$^{neo/+}$ (brown; two-tailed t-test, p=0.023, t=inf). No differences between Wt and *Mbp*$^{neo/+}$ (two-tailed t-test, p=0.27, t=2.496) **G)** Axon caliber distribution was not changed in *Mbp*$^{neo/neo}$ animals (orange; two-tailed Kruskal-Wallis F(2,69)=0.03, p=0.98 for both comparisons between Wt (black) and *Mbp*$^{neo/+}$ (brown) and Wt and *Mbp*$^{neo/neo}$).Mean and S.E.M.; data points are individual animals (B-C), and individual axon cross-sections (E-G). Source data are provided as a Source Data file.

Figure S2

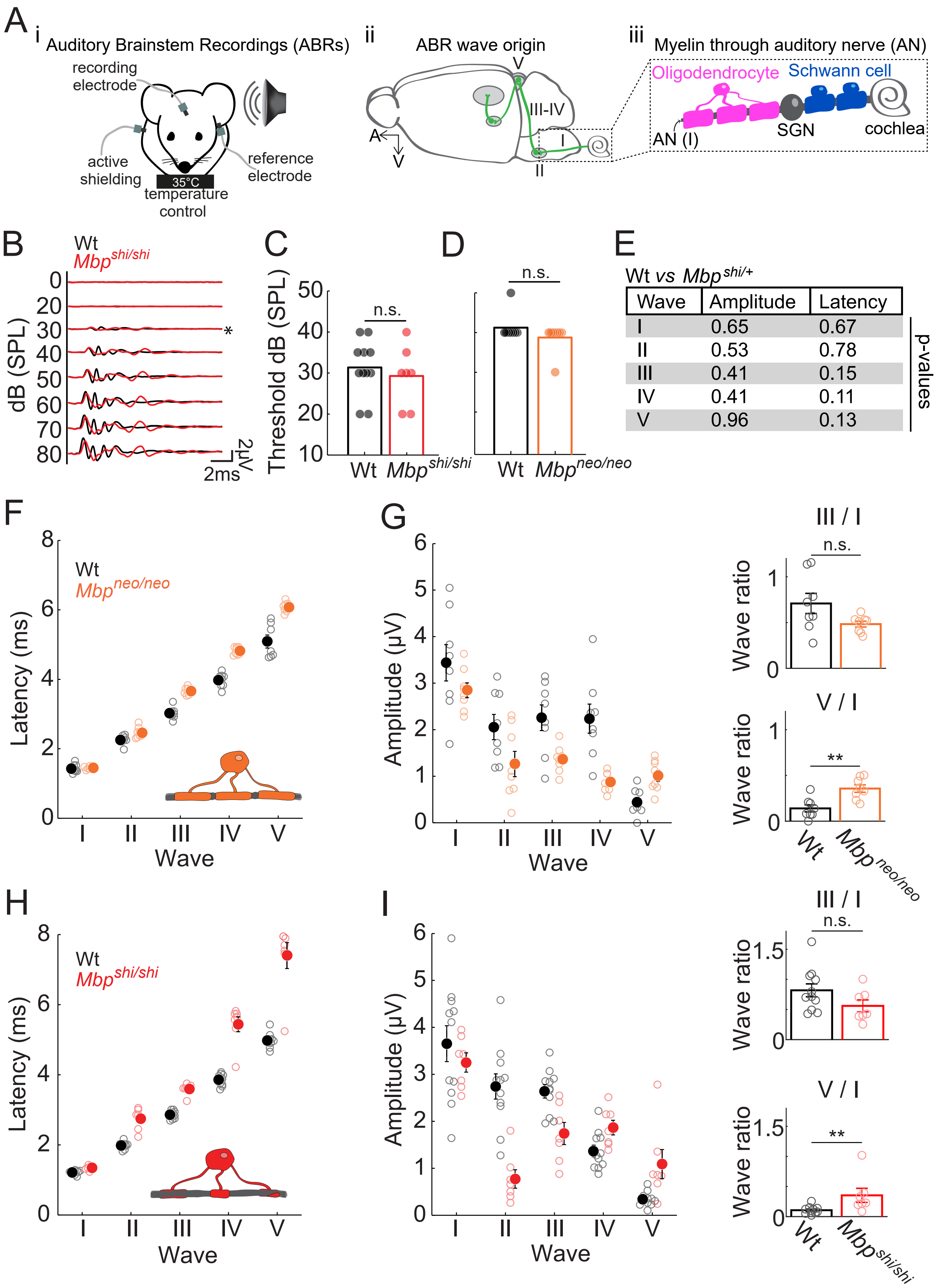
A
i Auditory Brainstem Recordings (ABRs)
recording electrode
active shielding
reference electrode
35°C
temperature control
ii ABR wave origin
V
III-IV
I
II
A
V
iii Myelin through auditory nerve (AN)
Oligodendrocyte
Schwann cell
AN (I)
SGN
cochlea
B
Wt
Mbp shi/shi
dB (SPL)
0
20
30
40
50
60
70
80
*
2μV
2ms
C
Threshold dB (SPL)
n.s.
Wt
Mbp shi/shi
D
n.s.
Wt
Mbp neo/neo
E
Wt vs Mbp shi/+
Wave | Amplitude | Latency
I | 0.65 | 0.67
II | 0.53 | 0.78
III | 0.41 | 0.15
IV | 0.41 | 0.11
V | 0.96 | 0.13
p-values
F
Latency (ms)
Wt
Mbp neo/neo
Wave
I II III IV V
G
Amplitude (μV)
Wave
III / I
n.s.
Wave ratio
V / I
**
Wt
Mbp neo/neo
H
Latency (ms)
Wt
Mbp shi/shi
Wave
I
Amplitude (μV)
Wave
III / I
n.s.
Wave ratio
V / I
**
Wt
Mbp shi/shi

Figure S2. CNS dysmyelination increased ABR latency and amplitude, but not hearing thresholds. **Ai)** Recording diagram. **Aii)** ABR peak origin. **Aiii)** Auditory nerve (AN) peripheral and central myelination; SGN: spiral ganglion neurons. **B)** Mean ABRs potentials per intensity for control (black; n=11) and *Mbp*$^{shi/shi}$ (red; n=7). *: sound detection threshold. **C)** Normal hearing thresholds in *Mbp*$^{shi/shi}$ (red; two-tailed Wrst, p=0.62, t=-0.7511) and **D)** in Mbp$^{neo/neo}$ (orange; n=8) (two-tailed Wrst, p=0.53, t=-2) against Wt (black; n=8). **E)** Normal 80dB amplitudes/latencies justifying combined control (7 *Mbp*$^{+/+}$ and 4 *Mbp*$^{shi/+}$). **F)** Increased peak latencies in *Mbp*$^{neo/neo}$ (orange) with group (two-way ANOVA, F(1,69)=147.62, p=8.32*10^{-19}), wave (two-way ANOVA, F(4,69)=1078.37, p=2.21*10^{-61}), and interaction effect (two-way ANOVA, F(4,69)=17.55, 5.63*10^{-10}). **G)** Amplitude difference in *Mbp*$^{neo/neo}$, with group (two-way ANOVA, F(1,70)=17.51, p=0.0001), wave (two-way ANOVA, F(4,70)=28.17, p=5.94*10^{-14}), and interaction effect (two-way ANOVA, F(4,70)=4.8, p=0.0018). Auditory gain (amplitude ratio) was comparable for waves III/I (upper right; Wrst, p=0.083, t=-7.03), and increased for waves V/I (lower right; Wrst, p=0.0047, t=5.285) suggesting compensatory gain increase. **H)** Latency in *Mbp*^{shi} (red) with group (two-way ANOVA, F(1,80)=235.63, p= 1.46*10^{-25}), wave (two-way ANOVA, F(4,80)=549.82, p=2.56*10^{-57}), and interaction effect (two-way ANOVA, F(4,80)=29.52, p=4.40*10^{-15}). **I)** Amplitude in *Mbp*$^{shi/shi}$ mice. Significant group (two-way ANOVA, F(1,80)=7.22, p=0.009), wave (two-way ANOVA, F(4,80)=35.17, p=6.23*10^{-17}) and interaction effect (two-way ANOVA, F(4,80)=10.67, p=5.55*10^{-07}). Wave V increases in *Mbp*$^{shi/shi}$ (lower right; Wrst, p=0.006, t=2.109) but not wave III (upper right; Wrst, p=0.085, t=-2.608). Wrst: Wilcoxon rank sum test. Mean and S.E.M.; data points are individual animals. Source data are provided as a Source Data file.

Figure S3

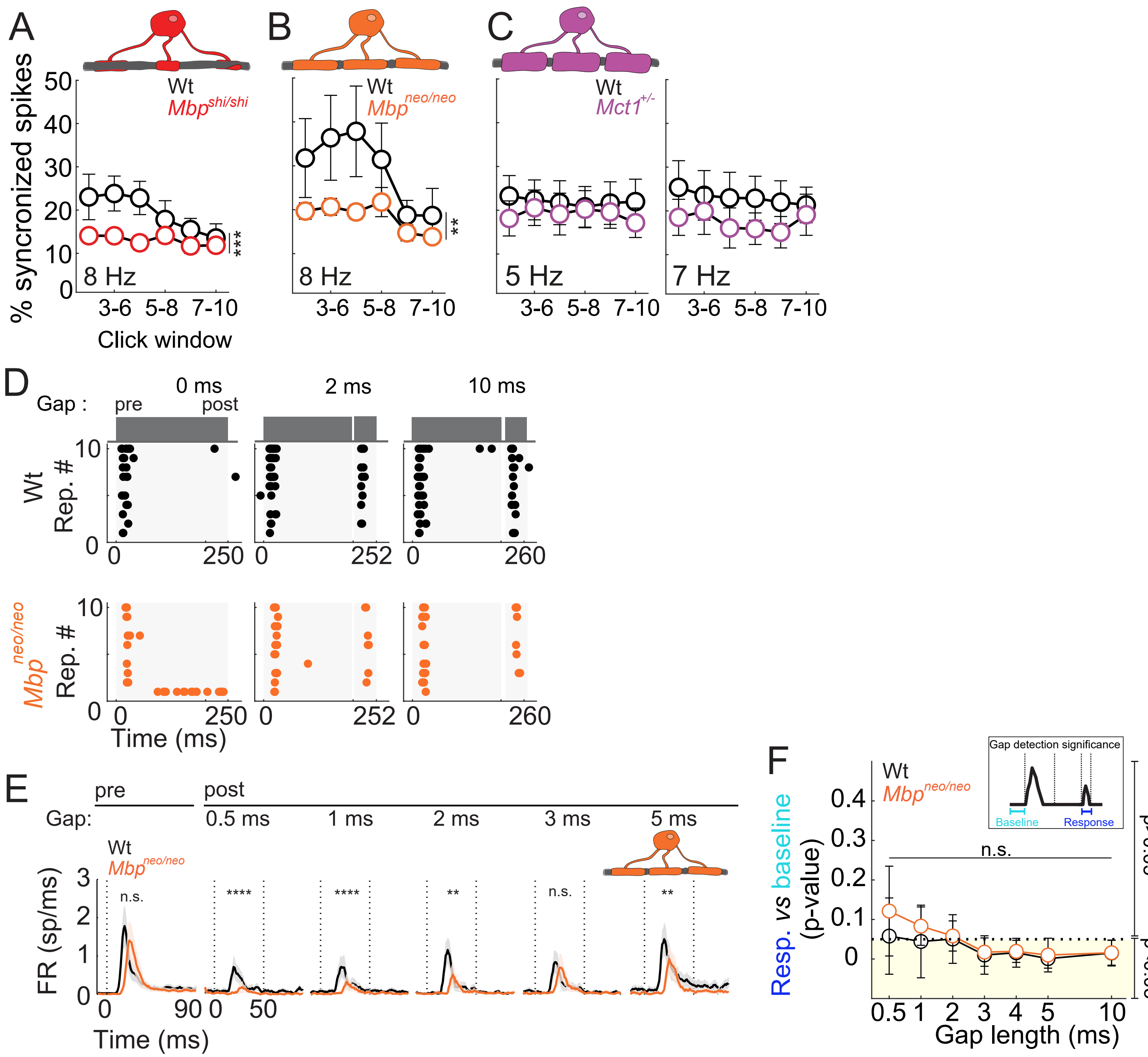

A
B
C
Wt
Mbp shi/shi
Mbp neo/neo
Mct1 +/-
% syncronized spikes
50
40
30
20
10
0
8 Hz
8 Hz
5 Hz
7 Hz
3-6
5-8
7-10
Click window
***
**
D
Gap :
pre
post
0 ms
2 ms
10 ms
Wt
Rep. #
Mbp neo/neo
250
252
260
Time (ms)
E
pre
post
Gap:
0.5 ms
1 ms
2 ms
3 ms
5 ms
FR (sp/ms)
n.s.
****
**
Time (ms)
F
Gap detection significance
Baseline
Response
Resp. vs baseline
(p-value)
p>0.05
p<0.05
Gap length (ms)

Figure S3. Temporal reliability and acuity are affected with dysmyelination and a loss of oligodendrocyte metabolic axonal support.

A-C) Same as in Figure 2E. **A)** *Mbp*$^{shi/shi}$ show a significant decrease in spike synchronicity at 8 Hz (red, n=6; Wt: n=3; one-way ANOVA, F(1,45)=16.95, p=0.0002), similar to **B)** *Mbp*$^{neo/neo}$ (orange, n=8; Wt: n=5; one-way ANOVA, F(1,60)=11.03, p=0.0015). **C)** No significant difference between *Mct1*$^{+/-}$ mice (purple; n=5 mice), and Wt (black; n=8 mice) at 5 Hz (left, one-way ANOVA, F(1,75)=1.06, p=0.30) or 7 Hz (same n as 5Hz; right, one-way ANOVA, F(1,69)=3.01,p=0.087). **D)** Same as in Figure 3B but for a *Mbp*$^{neo/neo}$ mouse (orange) and a Wt mouse (black). **E)** Same as in Figure 3D but for a *Mbp*$^{neo/neo}$ mice (orange; n=8) and their respective Wt (black; n=7). Significant effect of group was seen for most post-gap responses but not for pre-gap responses (two-way ANOVAs, [F(1,273)=0.27, p=0.60], [F(1,313)=33.67, p=$1.6*10^{-8}$], [F(1,313)=19.37, p=$1.48*10^{-5}$], [F(1,313)=10.2, p=0.0015], [F(1,313)=1.71, p=0.19, and [F(1,313)=8.93, p=0.003]; pre-gap and 0.5 to 5 ms post-gap respectively). **F)** Same as in Figure 3E but for the *Mbp*^{neo} line. No significant effect of gap was observed between Wt (black; n=22 recording sites, 7 mice) and *Mbp*$^{neo/neo}$ (orange; n=17 recordings, 8 mice) (Kruskal-Wallis, F(1,238)=0.92, p=0.337). E) Lines are averages across animals and shaded-error bars are S.E.M. F) Circles show the median per group per gap length, and error bars the S.E.median. Source data are provided as a Source Data file.

Figure S4

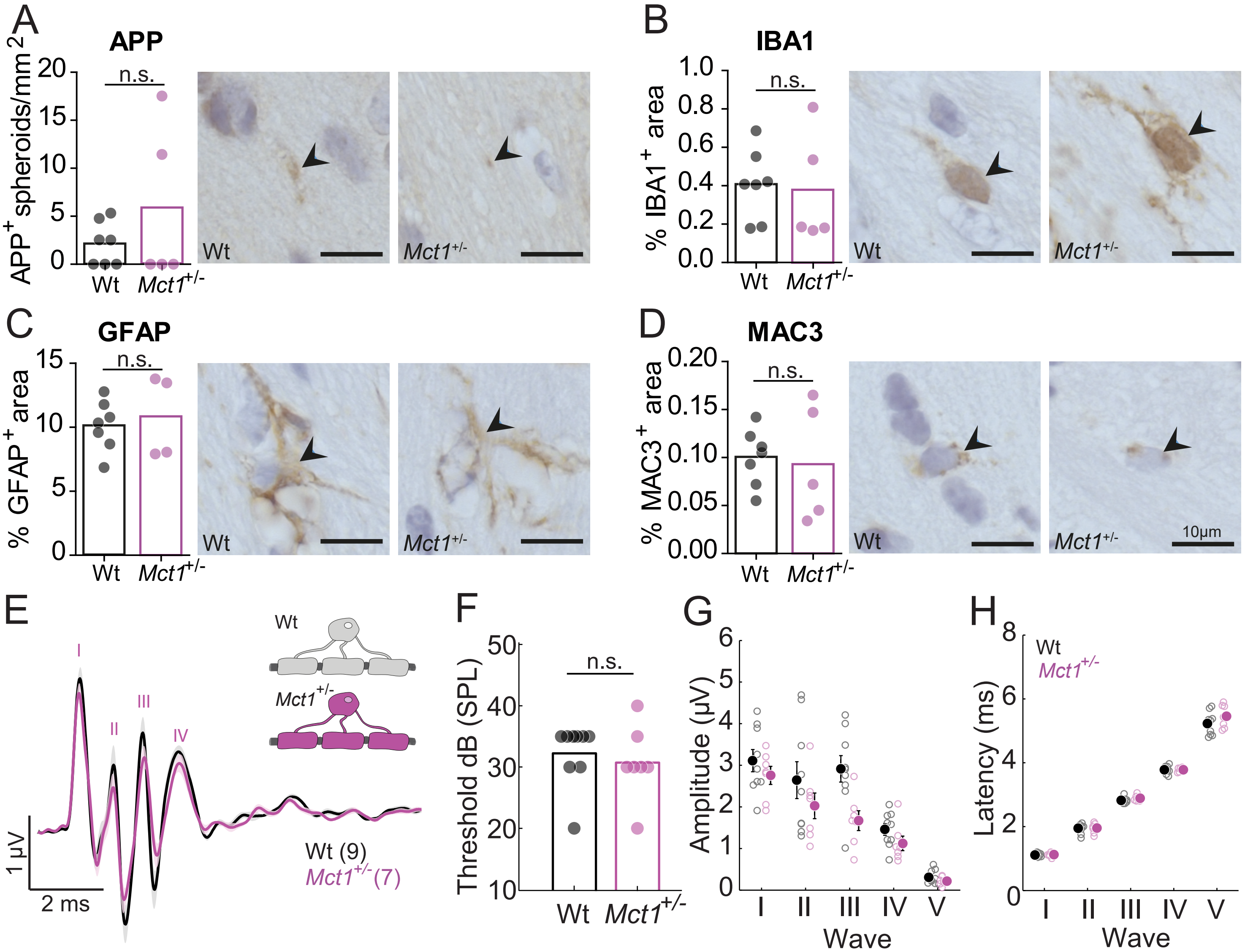

A
APP
n.s.
APP+ spheroids/mm2
20
15
10
5
0
Wt
Mct1+/-
B
IBA1
n.s.
% IBA1+ area
1.0
0.8
0.6
0.4
0.2
0.0
C
GFAP
n.s.
% GFAP+ area
D
MAC3
n.s.
% MAC3+ area
0.20
0.15
0.10
0.05
0.00
10µm
E
I
II
III
IV
1 µV
2 ms
Wt (9)
Mct1+/- (7)
F
n.s.
Threshold dB (SPL)
G
Amplitude (µV)
Wave
H
Latency (ms)
V

Figure S4. No evidence of neuronal degeneration in *Mct1*$^{+/-}$ mice.

Corpus callosum immunohistological quantifications of **A)** Amyloid precursor protein (APP) as a marker of axonal swellings, a feature of early neurodegeneration. No changes between groups (black Wt mice; purple *Mct1*$^{+/-}$ mice; two-tailed t-test, p=0.28, t=0.987). **B)** IBA1, a marker for activated microglia. No difference between groups (two-tailed t-test, p=0.83, t=-0.233). **C)** GFAP^{+}, a marker for astrocytes and astrogliosis. No change (two-tailed t-test, p=0.66, t=0.422). **D)** MAC3^{+}, a marker of commonly activated microglia or macrophages. No change (two-tailed t-test, p=0.78, t=-0.276). A-D) Wt mice n=7, and *Mct1*$^{+/-}$ mice =4-5. Specific labeling is in brown and nuclei were counterstained (blue). Black arrows show positive axons or cells that were quantified. Scale bar 10µm. **E)** Mean ABR response elicited by clicks at 80 dB in Wt (black) and *Mct1*$^{+/-}$ mice (purple) were comparable in shape and latency. **F)** Auditory thresholds were not different between groups (two-tailed Wilcoxon rank sum test, p=0.39, t=-0.657). **G)** Amplitude of waves I-V, revealed an effect of group (two-way ANOVA, F(1,70)=10.15, p=0.002) and, as expected, wave (two-way ANOVA, F(4,70)=32.21, p=3.2*10^{-15}), with no interaction (two-way ANOVA, F(4,70)=1.41, p=0.24). Multiple comparisons with Bonferroni correction revealed a close to significant difference for wave III (p=0.058) but not for waves I-II, IV-V (p>0.9 for all) **H)** Latency of waves I-V elicited by clicks at 80 dB was comparable between groups overall (two-way ANOVA, F(1,70)=2.09, p=0.15) and as expected, a significant difference was observed along the five waves (two-way ANOVA, F(4,70)=1179.93, p=2.07*10^{-63}), with no significant interaction (two-way ANOVA, F(4,70)=1.04, p=0.39). All graphs depict the mean and S.E.M. and individual data points are individual animals. E-H) Wt (black; n=9) and *Mct1*$^{+/-}$ mice (purple; n=7). Source data are provided as a Source Data file.

Figure S5

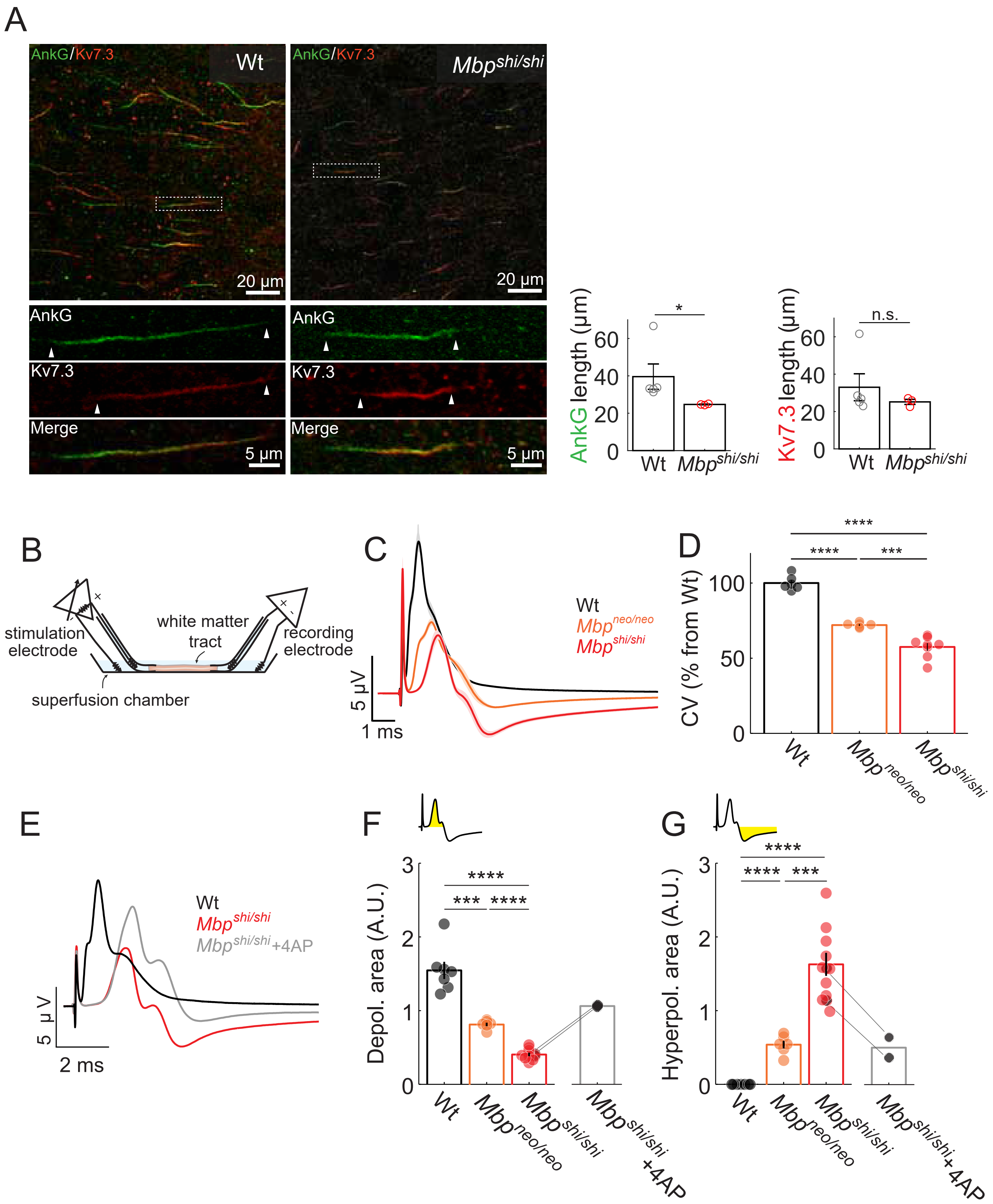
A
AnkG/Kv7.3
Wt
Mbpshi/shi
20 µm
AnkG
Kv7.3
Merge
5 µm
AnkG length (µm)
Kv7.3 length (µm)
60
40
20
0
*
n.s.
Wt
B
stimulation electrode
white matter tract
recording electrode
superfusion chamber
C
Wt
Mbpneo/neo
5 µV
1 ms
D
****
***
CV (% from Wt)
100
50
E
Mbpshi/shi+4AP
2 ms
F
Depol. area (A.U.)
3
2
1
+4AP
G
Hyperpol. area (A.U.)

Figure S5. White matter abnormalities and axonal initial segment (AIS) shifts resulting from dysmyelination

A) Stainings of AnkyrinG (green) and $K_v7.3$ channels (red) in the auditory cortex of Wt (left) and *Mbp*$^{shi/shi}$ (right). Below: magnifications of AIS. Right: reduction in AnkG length in *Mbp*$^{shi/shi}$ (red, two-sided Wilcoxon rank sum test, p=0.035, t=-73.597) but not $K_v7.3$ (right; two-sided Wilcoxon rank sum test, p=0.57, t=-5.747). For display background fluorescence (ROI without AIS) was subtracted. Wt n=5, 20-55 AIS/mouse. *Mbp*$^{shi/shi}$ n=3, 20-30 AIS/mouse. **B)** Scheme of optic nerve (ON) *ex vivo* recordings. **C)** Mean compound action potentials (CAP) from Wt, *Mbp*$^{shi/shi}$ and *Mbp*$^{neo/neo}$; stimulation current: 0.7 mA. Typical shape in Wt and *Mbp*$^{neo/neo}$, no triphasic shape in *Mbp*$^{shi/shi}$. **D)** Nerve conduction velocity at 0.7 mA reduced in *Mbp*$^{shi/shi}$ (two-tailed t-test, p=2.9*10^{-8}, t=-18.883), and *Mbp*$^{neo/neo}$ (two-tailed t-test, p=1.01*10^{-7}, t=-41.746). Reduction stronger in *Mbp*$^{shi/shi}$ (t-test, p=0.00038, t=-6.465). Wt (black): n=6 ON/6 mice; *Mbp*$^{neo/neo}$ (orange): n=6 ON/3 mice; *Mbp*$^{shi/shi}$ (red): n=8 ON/7 mice. **E)** Addition of 25µM 4-AP recovers partially CAP shape. **F)** Depolarizing CAP area (yellow) was reduced in both *Mbp*$^{neo/neo}$ (t-test, p=0.00013, t=-31.227) and *Mbp*$^{shi/shi}$ (t-test, p<0.0001, t=-48.693) and between *Mbp*$^{neo/neo}$ and *Mbp*$^{shi/shi}$ (two-tailed t-test, p=1.63*10^{-8}, t=-17.378). Lines for *Mbp*$^{shi/shi}$ before and after 4-AP. **G)** Hyperpolarizing CAP area (yellow) increased in *Mbp*$^{neo/neo}$ (two-tailed t-test, p=2.6*10^{-7}, t=10.189) and *Mbp*$^{shi/shi}$ (two-tailed t-test, p=3.17*10^{-7}, t=10.462) and *Mbp*$^{neo/neo}$ and *Mbp*$^{shi/shi}$ (t-test, p=0.00012, t=7). A recovery of depolarization/hyperpolarization area observed in the presence of 4-AP (gray). E-G) Wt: n=7 ON/7 mice; *Mbp*$^{neo/neo}$: n=6 ON/3 mice; *Mbp*$^{shi/shi}$: n=10 ON/9 mice; 4-AP treated *Mbp*$^{shi/shi}$: n=2 ON/2 mice. Graphs show means, error-bars/shadows S.E.M., and data points depict individual ON. Source data are provided as a Source Data file.

Figure S6

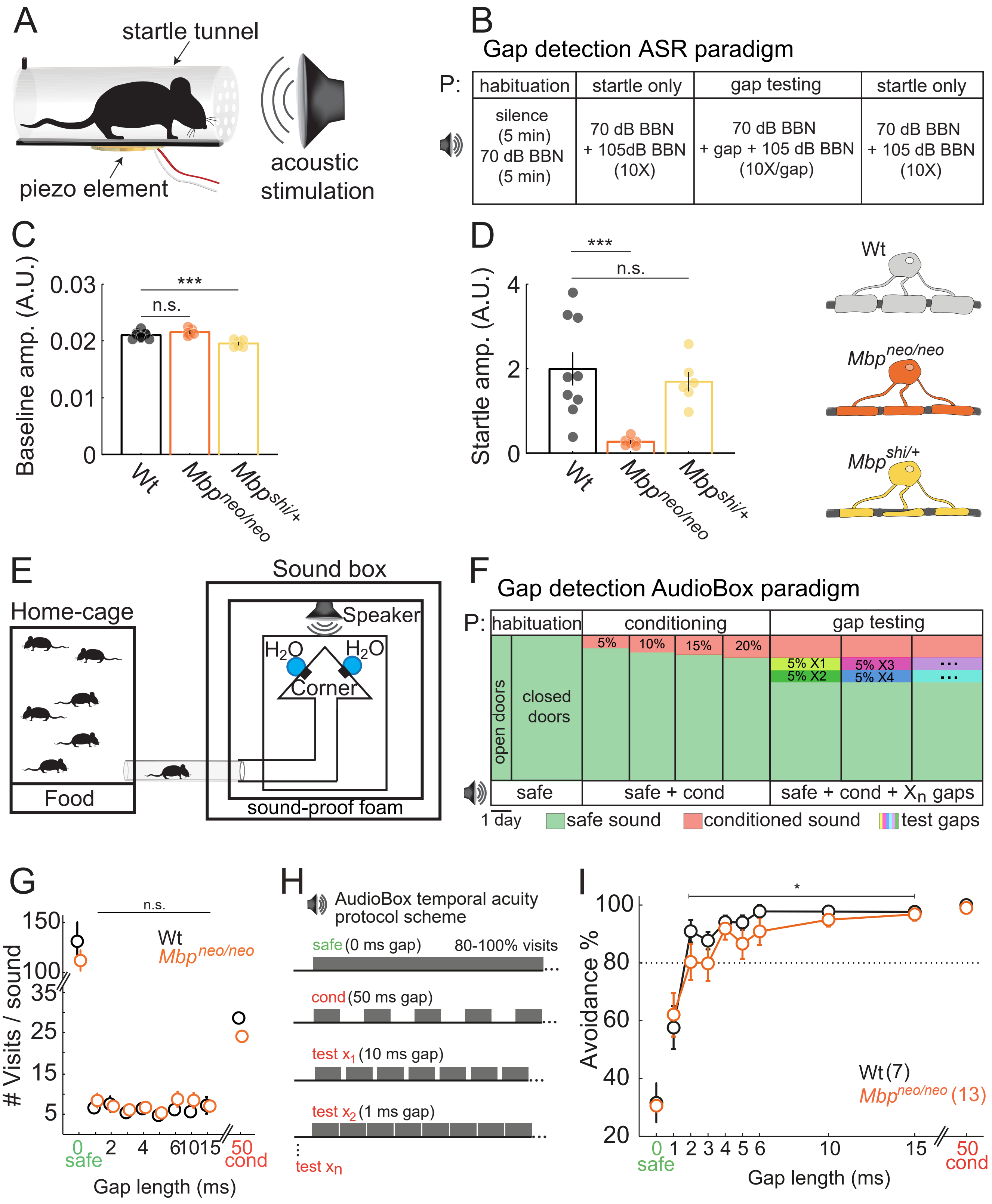
A
startle tunnel
piezo element
acoustic stimulation
B
Gap detection ASR paradigm
P:
habituation
startle only
gap testing
startle only
silence (5 min) 70 dB BBN (5 min)
70 dB BBN + 105dB BBN (10X)
70 dB BBN + gap + 105 dB BBN (10X/gap)
70 dB BBN + 105 dB BBN (10X)
C
Baseline amp. (A.U.)
0.03
0.02
0.01
0
n.s.
***
Wt
Mbpneo/neo
Mbpshi/+
D
Startle amp. (A.U.)
4
2
0
***
n.s.
Wt
Mbpneo/neo
Mbpshi/+
Wt
Mbpneo/neo
Mbpshi/+
E
Home-cage
Food
Sound box
Speaker
H2O
H2O
Corner
sound-proof foam
F
Gap detection AudioBox paradigm
P:
habituation
conditioning
gap testing
open doors
closed doors
5%
10%
15%
20%
5% X1
5% X2
5% X3
5% X4
safe
safe + cond
safe + cond + Xn gaps
1 day
safe sound
conditioned sound
test gaps
G
Visits / sound
150
100
35
25
15
5
n.s.
Wt
Mbpneo/neo
0 safe
2
4
6
10
15
50 cond
Gap length (ms)
H
AudioBox temporal acuity protocol scheme
safe (0 ms gap)
80-100% visits
cond (50 ms gap)
test x1 (10 ms gap)
test x2 (1 ms gap)
test xn
I
Avoidance %
100
80
60
40
20
*
Wt (7)
Mbpneo/neo (13)
0 safe
1 2 3 4 5 6
10
15
50 cond
Gap length (ms)

Figure S6. Behavioral temporal acuity testing protocols.

A) Schematic of gap-detection inhibition of auditory startle reflex (GDIASR) test. **B)** Diagram of the GDIASR sound protocol. **C)** Baseline movement comparable between Wt (black; n=9) and $Mbp^{neo/neo}$ mice (orange; n=6) (t-test, p=0.14, t=2.095). Reduction in baseline movement in $Mbp^{shi/+}$ (yellow; n=6; two-sided Wilcoxon rank sum test, p=0.00039, t=-5.614). **D)** Amplitude of startle-only response (5 largest trials of the initial 10 ASR-only trials) was lower in $Mbp^{neo/neo}$ compared to Wt (two-sided Wilcoxon rank sum test, p=0.00079, t=-41.885), but not different between Wt and $Mbp^{shi/+}$ (two-sided Wilcoxon rank sum test, p=0.86, t=-1.393). **E)** AudioBox diagram. Mice live in the home-cage with access to food and enter the 'sound box' corner for water. **F)** Diagram of the AudioBox gap-detection paradigm with three main phases: habituation (continuous BBN), conditioning (BBN with 50ms gaps) and gap testing (introduction of BBN with gaps>0ms and <50ms). **G)** Mean visits per sound for the duration of the experiment for Wt (black, n=7) and $Mbp^{neo/neo}$ (orange, n=13) animals. No differences between groups were seen for the overall sounds analyzed (two-way ANOVA, F(1,144)=1.91, p=0.17). **H)** Examples of sounds used in AudioBox paradigm. Safe visits to the corner were accompanied by safe sound (continuous BBN, ~70 dB), and water was available. Conditioned visits were accompanied by the conditioned sound (cond; BBN with 50 ms gaps), water was not available, and air-puff was delivered upon nose-poke. Once animals discriminated safe and conditioned, test visits with test sounds (gaps > 0 ms and <50 ms) were introduced. Water was available in these visits. **I)** Avoidance behavior (proportion of visits without nose-pokes) revealed a reduction (two-way ANOVA, F(1, 126)=4.81, p=0.030) in $Mbp^{neo/neo}$ animals (orange; n=13) for gaps >1 ms compared to Wt (black; n=7) confirming a behavioral deficit in temporal acuity in a naturalistic environment. All graphs depict the mean and error bars S.E.M. Source data are provided as a Source Data file.

Figure S7

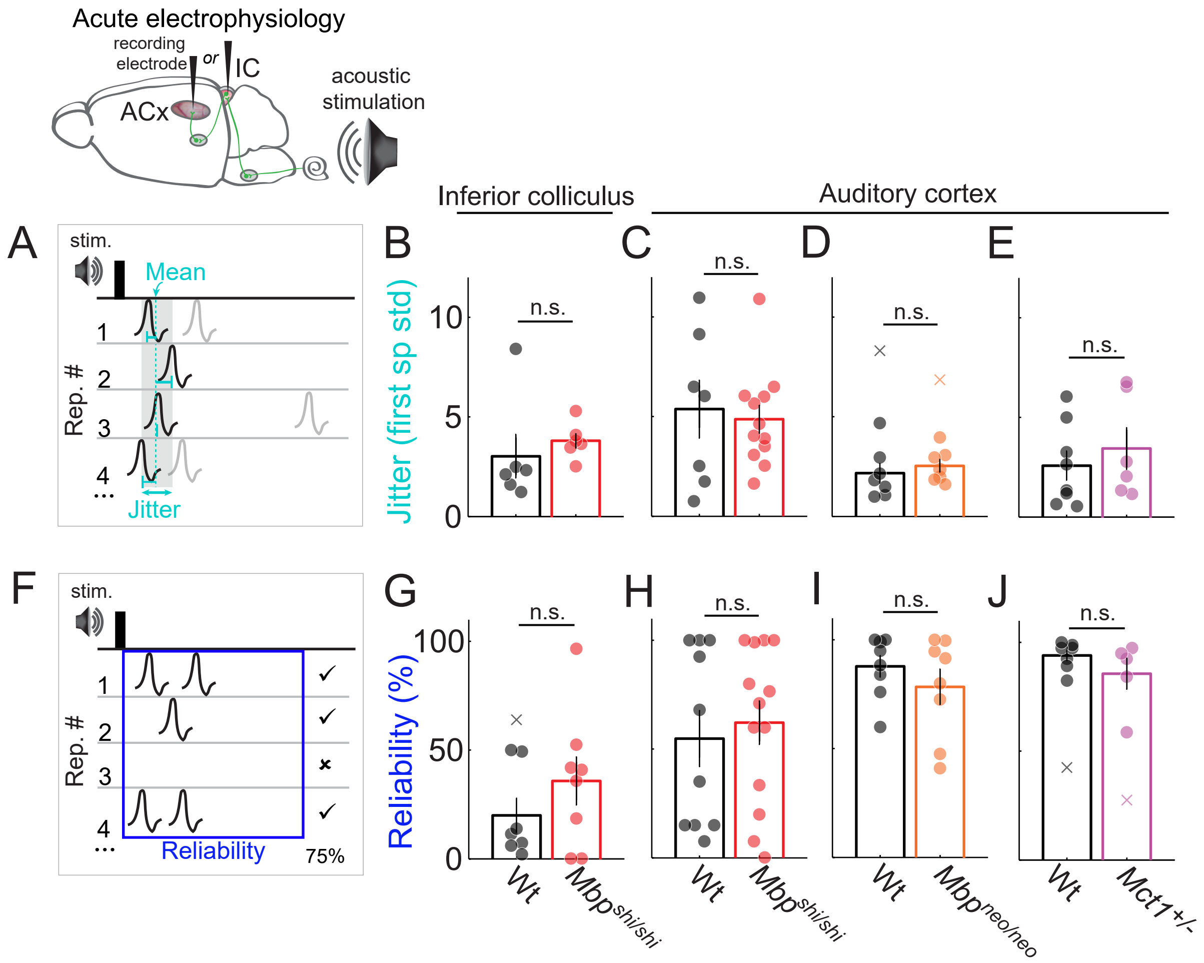
Acute electrophysiology
recording electrode
or
IC
ACx
acoustic stimulation
Inferior colliculus
Auditory cortex
A
B
C
D
E
stim.
Mean
Rep. #
1
2
3
4
Jitter
Jitter (first sp std)
10
5
0
n.s.
F
G
H
I
J
Reliability
75%
Reliability (%)
100
50
Wt
Mbp shi/shi
Mbp neo/neo
Mct1+/-

Figure S7. Jitter and reliability are overall not affected with dysmyelination or an axo-glial metabolic defect.

A) Schematic of the jitter measurement. Jitter was defined as the standard deviation of the first spike latency/repetition. **B-E)** Jitter was normal in all mutant mice. Jitter was unchanged in *Mbp*$^{shi/shi}$ animals (red, n=6) compared to Wt (black, n=6) **B)** in IC (two-sided Wilcoxon rank sum test, p=0.065, t=2.42) and **C)** in ACx (Wt: n=7; *Mbp*$^{shi/shi}$: n=12, two-sided Wilcoxon rank sum test, p=0.90, t=-0.753). **D)** Jitter was unchanged in ACx of *Mbp*$^{neo/neo}$ mice (orange, n=8) compared to Wt (black, n=8) (two-sided Wilcoxon rank sum test, p=0.26, t=1.183) and **E)** in ACx of *Mct1*$^{+/-}$ mice (purple, n=6) compared to Wt (black, n=8) (two-sided Wilcoxon rank sum test, p=0.41, t=0.822). **F)** Schematic of the measurement of spiking reliability: the percentage of repetitions that elicited at least one spike within a 50 ms window. **G-J)** Reliability was normal in all mutant mice. Reliability was normal in *Mbp*$^{shi/shi}$ animals (red, n=8)) compared to Wt (black, n=7) **G)** in IC (two-sided Wilcoxon rank sum test, p=0.51, t=1.427), and **H)** in ACx (two-sided Wilcoxon rank sum test, p=0.80, t=0.729; Wt: n=10; *Mbp*$^{shi/shi}$: n=13). **I)** Reliability was also normal in ACx of *Mbp*$^{neo/neo}$ mice (two-sided Wilcoxon rank sum test, p=0.44, t=-1.154; Wt: n=8; *Mbp*$^{neo/neo}$: n=8), and **J)** in ACx of *Mct1*$^{+/-}$ mice (two-sided Wilcoxon rank sum test, p=0.28, t=-1.188; Wt: n=7; *Mct1*$^{+/-}$: n=5). All graphs depict the mean and S.E.M. and individual data points are individual animals. Outliers are depicted with an ‘x’ and were not considered in the statistical analysis. Source data are provided as a Source Data file.

Supplementary Methods

Quantitative RT-PCR

Half a hemisphere of 8-week-old *Mbp*$^{neo/neo}$ and *Mbp*$^{neo/+}$ mice and littermate controls were homogenized in TRIzol (Invitrogen) using Polytron PT 3100 (Kinematica, Luzern, Switzerland). RNA was extracted and purified using RNeasy Columns (Quiagen). The quality and integrity of purified RNA was secured using the Agilent 2100 Bioanalyser (Agilent Technologies, Santa Clara, U.S.A). cDNA was synthesized using random nonamer primers, oligo dT primers and the SuperScript III RNA H Reverse Transcriptase (Invitrogen). Quantitative RT-PCR was performed using the 7500 Fast Real-Time PCR system (Applied Biosystems, Foster City, U.S.A). The Power SYBR Green PCR Master Mix (Applied Biosystems, Foster City, U.S.A) was used. Reactions were performed in quadruplicates. The PCR reaction was carried out using the following program: 60°C for 1 min and 95°C for 15 sec for 45 cycles. The abundance of the transcripts was analyzed in relation to the mean of the standards Rps13 and Cyclophyllin A, which both did not differ between genotypes. Primers sequences specific for MBP can be found in the Supplementary List of Primers.

Western Blotting

A myelin enriched membrane fraction was purified from mouse brains lysates as described in[1]. For the *Mbp*^{neo} mouse line, 8-week-old littermates were used. The protein concentration of lysates and the myelin enriched fraction was determined using the DC protein assay (BioRad, Hercules, U.S.A). Proteins were blotted onto polyvinylidene difluoride membranes (Roche, Basel, Switzerland), blocked with 5% milk powder in TBS/0.05% Tween-20 and incubated with primary antibodies diluted in the same blocking solution. For *Mbp*^{neo} line: rabbit anti-MBP (1:500, Dako A0623, Santa Clara**,** U.S.A) was incubated overnight at 4°C. Proteins on PVDF were washed with TBS/0.05% Tween-20. The secondary antibody HRP goat anti rabbit (1:10000, Dianova, Hamburg, Germany) was used. Immunoblots were scanned (Intas ChemoCam system, Göttingen, Germany) and processed with Adobe Photoshop.

Electron microscopy

G-ratios (axonal diameter/fiber diameter) were determined by analyzing 100 axons per animal selected by systematic random sampling on 10 random images taken at a magnification of 8,000 x. For optic nerve EM of the *Mbp*$^{neo/neo}$ mouse line, 3 mice per genotype (8 weeks old) were used. Optic nerves were carefully dissected and fixed by immersion into the previously mentioned fixing solution. The samples where embedded and polymerized in Epon (Serva) after postfixation as

shown in[2]. Ultrathin sections were placed on Formvar-coated grids (Science Services, Munich, Germany) and stained for 30 min with 4% Uranylacetat and 6 min Leadcitrate after Reynolds[3]. Transmission EM was conducted and 10 random non-overlapping images were taken per optic nerve at 7000× magnification using a Zeiss 900 transmission electron microscope (Zeiss, Oberkochen, Germany) equipped with 2 K CCD Camera Sharp-eye (Troendle, Moorenweis, Germany). Electron micrographs were analyzed using ImageJ (NIH, Bethesda, U.S.A). g-Ratios were determined as the ratio between the axonal feret diameter and the feret diameter of the corresponding myelin sheath. For randomization, a regular grid of 2.5 µm node to node distance was placed on the picture. All axons crossed by the grating were assessed, yielding a minimum of 500 myelinated axons per animal.

For quantification of proportion of non-myelinated axons, all axons on the micrographs (a minimum of 600 axons per animal) were categorized as either myelinated or unmyelinated axons. Axons were counted as myelinated if ensheathed by at least one complete layer of compacted myelin and the absence of uncompact myelin. For quantification of axonal densities, axons crossing the image borders were not considered. All quantifications were performed blinded to the genotype. Statistical analysis of quantification of myelinated and non-myelinated fibers and g-ratios was evaluated with two-sided Student-T-tests. Differences in axon caliber distributions were evaluated with two-sample Kolmogorov-Smirnov tests.

ABRs

Hearing thresholds were determined as the lowest intensity that evoked a response that was 3 times the root mean square of the response at 0 dB within a window of 1 ms centered on wave I. In addition, this measurement was confirmed by visual inspection of the lowest intensity that evoked a response (compared to 0 dB) within 6 ms from stimulus onset. The identity of the waves was determined by visual selection of the maximum peak value corresponding to each of the five waves according to the typical reported position of each wave (1 ms apart) on the averaged 1000 repetitions per stimuli. After manual selection of each wave peak and trough, amplitude was measure as the peak to trough distance, and latencies as the time to each peak maximum. For the analysis, an ANOVA was used to assess the changes in latencies or amplitudes of each wave between groups.

Immunohistochemistry

Histological analysis was performed essentially as described[4]. 5-7 mice per genotype were analyzed at an age of 12 to 16 weeks. Antibodies were specific for IBA1 (019-19741, Wako,

1:1000), amyloid precursor protein APP (MAB348, Millipore, 1:1000), glial fibrillary protein GFAP (Z0334, Dako, 1:200), MAC3 (553322, BD Pharmingen, 1:400) or CD3 (ab11089, abcam, 1:250). Images were obtained using a bright field microscope (Zeiss Imager.Z1) equipped with a Zeiss Plan-Neofluar 20x/ 0.50 objective and a Zeiss AxioCam MRc camera controlled by the Zeiss Zen Pro 1.0 software with 10% overlap. The software was also used for shading correction and stitching of the tiles. Images were quantified using Image J 1.50e. For quantification, the hippocampal fimbria and corpus callosum were selected. APP positive spheriods and number of CD3-positive T-cells are given as number per square millimeter. For quantification of GFAP-, MAC3- or IBA1 positive area, an ImageJ macro was used[5]. All quantifications were performed blinded to the genotype. For statistical analysis a two-tailed t-test was performed. For representative figures, images were obtained using the Imager.Z1 equipped with a Zeiss AxioCam MRc camera and a Zeiss Plan-Neofluar 100x/ 1.3 oil immersion objective. Images were processed using Adobe Photoshop.

AIS stainings

Cutting solution contained (in mM): 125 NaCl, 25 $NaHCO_3$, 1.25 $NaH_2PO_4•H_2O$, 3 KCl, 25 glucose, 1 $CaCl_2$, 6 $MgCl_2$, 1 kynurenic acid Blocking solution contained 5% goat serum and 0.5% Triton X-100 in PBS. Primary antibodies used were Anti-Kv7.3 (KCNQ3n) raised in guinea pig (1:200) kindly provided by Ed Cooper (Baylor College of Medicine, Houston, TX, USA). AnkyrinG antibody was raised in rabbit (1:100; Santa Cruz). Secondary antibodies: Alexa 488-conjugated donkey anti-rabbit (1:1000, Invitrogen) and Cy3 donkey anti-guinea pig, (1:1000, Dianova). Mice were euthanized by cervical dislocation, the brain removed, submerged in ice-cold cutting solution and coronal 300 µm slices were prepared using a vibratome (VT1000S, Leica) with cutting amplitude of 1 and 0.05-0.07 forward speed. Freshly cut slices were fixed with Methanol chilled at -20°C after removal of the cutting solution and incubated at -20°C for 10 min. The slices were washed with PBS 1X (3 times, 5 min. each) and blocked for 2.5-5 hours at room temperature. The primary antibodies dissolved were added after blocking and slices incubated for 2 days at room temperature. Slices were washed with PBS 1X (3 times, 5 min. each), and secondary antibodies were added and left for 2 hours at room temperature (protected from the light). The slices were washed with PBS 1X (4 times, 5 min. each), stained with DAPI (4',6-diamidino-2-phenylindole) for 10min., and then washed with PBS 1X (4 times, 5min. each). Slices were mounted using Aqua-Poly/Mount (Polysciences, Inc) and stored at 4°C protected from the light.

AIS were imaged using a confocal microscope (Zeiss 510 meta). Z-stacks were acquired using a 40x objective (1 µm step, 30 z per stack in average). 5 fields were imaged per mouse. AIS length analysis was performed from confocal scans. The immuno-signal of AnkyrinG and Kv7 were measured with the segmented line tool in FIJI (v.1.51) by drawing a line along the AIS. For both channels the beginning and end of the expression was defined when the immuno- signal was stronger than the background. No background subtraction was applied. Comparisons were done for all AIS quantified per animal and using a t-test for comparison between Mbp^{shi} mutants and Wt mice.

Optic nerve recordings

Artificial cerebrospinal fluid (aCSF) was prepared, containing (in mM): 124 NaCl, 3 KCl, 2 $CaCl_2$, 2 $MgSO_4$, 1.25 NaH_2PO_4, 23 $NaHCO_3$ and 10 glucose monohydrate. The solution had a pH of 7.4 and was controlled for osmolarity. Both stimulating and recording suction electrodes were fabricated from borosilicate glass capillaries as previously described[6]. An Ag/AgCl wire was inserted into the electrode, and the capillary space back-filled with aCSF with 10 mM glucose.

Experiments were done as reported before[7,8]. Mice were decapitated and the skin over the skull was removed. The optic nerves were separated from the eyes at the ocular cavity. Then, the skull was opened, and the optic nerves detached by cutting caudally to the optic chiasm. The preparation was placed into an interface perfusion chamber (Harvard Apparatus, Holliston, MA) and continuously superfused with carbogen (95% O_2, 5% CO_2) bubbled aCSF at 37°C during the experiment at about 4 mL/min. Optic nerves were detached from the chiasm and placed in the suction electrodes for recording. The stimulation electrode was attached to the end that was near the retina. Continuous stimulation of 0.75 mA at 0.1 Hz was performed until the recorded compound action potential (CAP) showed a steady shape (typically between 10 and 40 min.). For stimulation, the electrode was connected to a battery (Stimulus Isolator 385; WPI, Berlin, Germany) that delivered a supramaximal stimulus to the nerve. The recording electrode was connected to an EPC9 amplifier (Heka Elektronik, Lambrecht/Pfalz, Germany). Signals were amplified 500 times, filtered at 30 kHz, and acquired at 100 kHz.

The CAP consists typically of 3 peaks of different amplitudes and latencies. Peak 1 is the fastest (approximately at 0.5 ms after stimulation) and it reflects the activity of high caliber myelinated axons Peak 2, at 1ms, reflects the activity of middle caliber axons, and peak 3 (1.5 ms) of small caliber axons. Since in $Mbp^{shi/shi}$ mice it was not possible to distinguish peak 1, the analysis was

done independently of peak specification. We measured the maximal amplitude elicited by specific stimulation and the correlated area under the curve. The CAP area is a reliable measure that reflects the proportion of axons that are activated by the stimulation. For these recordings, 6 to 10 nerves from 3 to 9 animals per group were used and pooled in the analysis.

CAP amplitude was measured as the maximum data point of the whole CAP curve. We believe the measurements mainly reflect the activity of middle caliber axons. For the detection of the depolarization amplitude, a window of 3.7 ms after the stimulation artifact was taken, to ensure detection of peaks even in the mouse models with delayed responses. For the quantification of the hyperpolarizing peak, a window of 3 ms around the maximum negative peak was taken. Statistical analysis of depolarization and hyperpolarization peaks was done using 2-way ANOVAs. For the measurement of conduction velocity, we obtained the latency to the maximum peak at a stimulation of 0.7 mA, and the length of the nerve stretched between the electrodes, which was measured after the end of every experiment. The latency/length value was normalized to the mean of conduction velocity values of the control animals. Parametric t-test was performed for statistical analysis.

AudioBox

The AudioBox system was used to test behavioral gap-detection. The AudioBox (New*Behavior*, TSE systems) is an automated system for behavioral acoustic conditioning[9–11]. This system is designed to minimize the interference from the experimenter and provides a spatially and socially enriched environment in which female mice live in group (up to 10 mice) for several days**.** The setup consisted of a home-cage, where animals had food *ad-libitum,* and a corner within a sound-attenuated box, where they could access water also *ad-libitum* (Figure S6E). A speaker (22TAF/G, Seas Prestige) was located over the corner. Animals carried a transponder (implanted under anesthesia 3 days before) that was detected by an antenna at the entrance of the corner. Time spent in the corner, number of nose-pokes and sound played were detected through sensors in the corner. For these experiments, *Mbp*$^{neo/neo}$ mice were chosen over *Mbp*$^{shi/shi}$ because of their normal life span and lack of motor impairments. Three experiment replications were pooled together. A final number of 7 control animals (Wt) and 13 mutant animals (*Mbp*$^{neo/neo}$) that were 6-14 weeks old at the start of the experiment were used.

Behavioral testing occurred in the corner. Each time a mouse entered the corner a “visit” started, and a sound was played for the duration of the visit. The training paradigm, summarized in Figure

S6F, consisted of 3 phases. During the habituation (one day doors opened, and 3-9 days doors closed), the safe sound (continuous BBN) was played in all visits. In this phase, animals could nose-poke and get access to water. During the conditioning phase ‘conditioned’ visits were introduced. In safe visits, as before, the animal could make a nose-poke and get water. Animals that did not nose-poke in more than 60% of safe visits were immediately excluded from the behavioral experiment. This was the case for 11 out of a total of 18 Wt and 7 out of 20 *Mbp*$^{neo/neo}$ tested. In conditioned visits (pseudo-randomly distributed according to probabilities as in Figure S6F), however, a conditioned sound (BBN interrupted by 50 ms silent gaps every 500 ms) was played, and a nose-poke triggered an air-puff, and no opening of the doors. Mice had to learn to not nose-poke in visits in which silent gaps interrupted the sound. Typically, this occurred 1-2 days after introduction of the conditioned visits. The percentage of conditioned sound presentation was increased stepwise 5% every 2 days. After the conditioning phase, the gap testing phase began. Every 4 days two new visit types were introduced, each with BBN sounds interrupted, like the conditioned sounds, by gaps, which varied in length with visit type (Figure S6F). During this phase 70% of visits were safe, 20% conditioned and 10% conditioned gaps. A total of 15 gaps were tested with lengths between 1 and 45 ms. The new sounds were chosen in pairs in a semi-random fashion, such that the more difficult gaps (gaps below 5 ms) were presented in the same block of days as gaps longer than 5 ms. A minimum of four days with each pair of sounds was allowed before starting a new gap testing.

To analyze sound exposure, the number of visits per sound for each animal was averaged within the first two days of exposure to specific gaps. The percentage of avoidance was quantified as the number of visits without nose-pokes for each sound played, only considering the first two days of training for each sound. The number of visits was compared to ensure equivalent sound exposure. The data from visits with gaps between 2 and 15 ms duration, for which wild type mice showed no impairment, were used for statistical comparison.

Acute electrophysiology

Reliability measurements were obtained from the same data used for the amplitude. The percentage of trials that contained at least one spike within a 37 ms window that started 12 ms after stimulus onset was obtained. Jitter was measured also under these conditions as the standard deviation from the mean 1st-spike latency value. All measurements were performed in single recording sites and then averaged for each animal. All comparisons were done using parametric or non-parametric t-tests.

List of Primers

qRT-PCR

Primer	Forward (5'-3')	Reverse (5'-3')
MBP	GCCTGTCCCTCAGCAGATT	GCCTCCGTAGCCAAATCC

Genotyping

Primer	Forward (5'-3')	Reverse (5'-3')
**Mbp^{neo} line**		
MBP WT	GGGTGATAGACTGGAAGGGTTG	GCTAACCTGGATTGAGCTTGC
Lar3 (LacZ)	-	CAACGGGTTCTTCTGTTAGTCC
**Mbp^{shi} line**		
MBP WT	GAGCTCTGGTCTTTTCTTGCAG	CCCGTGGTAGGAATATTACATTAC
Shi	CAGGGGATGGGGAGTCAG	ATGTATGTGTGTGTGTGCTTATCTAGTGTA
**Mct1 line**		
Mct1	GTGTCACCCAACACTGATAACAAGAG	TGATACTTCACTGGTCGTTGCA
ßGalRe61	-	GATTAAGTTGGGTAACGCCAG

References

1. Jahn, O., Tenzer, S. & Werner, H. B. Myelin proteomics: molecular anatomy of an insulating sheath. *Mol. Neurobiol.* **40,** 55–72 (2009).

2. Möbius, W. *et al.* Electron microscopy of the mouse central nervous system. *Methods Cell Biol.* **96,** 475–512 (2010).

3. Reynolds, E. S. The use of lead citrate at high pH as an electron-opaque stain in electron microscopy. *J. Cell Biol.* **17,** 208–212 (1963).

4. de Monasterio-Schrader, P. *et al.* Uncoupling of neuroinflammation from axonal degeneration in mice lacking the myelin protein tetraspanin-2. *Glia* **61,** 1832–1847 (2013).

5. Wieser, G. L. *et al.* Neuroinflammation in white matter tracts of Cnp1 mutant mice amplified by a minor brain injury. *Glia* **61,** 869–880 (2013).

6. Stys, P. K., Ransom, B. R. & Waxman, S. G. Compound action potential of nerve recorded by suction electrode: a theoretical and experimental analysis. *Brain Res.* **546,** 18–32 (1991).

7. Saab, A. S. *et al.* Oligodendroglial NMDA receptors regulate glucose import and axonal energy metabolism. *Neuron* **91,** 119–132 (2016).

8. Trevisiol, A. *et al.* Monitoring ATP dynamics in electrically active white matter tracts. *Elife* **6,** (2017).

9. de Hoz, L. & Nelken, I. Frequency tuning in the behaving mouse: different bandwidths for discrimination and generalization. *PLoS One* **9,** e91676 (2014).

10. Chen, C., Krueger-Burg, D. & de Hoz, L. Wide sensory filters underlie performance in memory-based discrimination and generalization. *PLoS One* **14,** e0214817 (2019).

11. Cruces-Solís, H. *et al.* Auditory midbrain coding of statistical learning that results from discontinuous sensory stimulation. *PLoS Biol.* **16,** e2005114 (2018).
