# Supplementary material for: A role of oligodendrocytes in information processing
Source: Nat Commun. 2020 Oct 30;11:5497. doi: 10.1038/s41467-020-19152-7 (PMC7599337; doi:10.1038/s41467-020-19152-7)
Supplement: Supplementary file 2 — Reporting Summary [file 41467_2020_19152_MOESM2_ESM.pdf]

nature research

Corresponding author(s): Livia de Hoz

Last updated by author(s): Sep 10, 2020

Reporting Summary

Nature Research wishes to improve the reproducibility of the work that we publish. This form provides structure for consistency and transparency in reporting. For further information on Nature Research policies, see our Editorial Policies and the Editorial Policy Checklist.

Statistics

For all statistical analyses, confirm that the following items are present in the figure legend, table legend, main text, or Methods section.

n/a	Confirmed	
☐	☒	The exact sample size (n) for each experimental group/condition, given as a discrete number and unit of measurement
☐	☒	A statement on whether measurements were taken from distinct samples or whether the same sample was measured repeatedly
☐	☒	The statistical test(s) used AND whether they are one- or two-sided<br>*Only common tests should be described solely by name; describe more complex techniques in the Methods section.*
☒	☐	A description of all covariates tested
☐	☒	A description of any assumptions or corrections, such as tests of normality and adjustment for multiple comparisons
☒	☐	A full description of the statistical parameters including central tendency (e.g. means) or other basic estimates (e.g. regression coefficient) AND variation (e.g. standard deviation) or associated estimates of uncertainty (e.g. confidence intervals)
☐	☒	For null hypothesis testing, the test statistic (e.g. F, t, r) with confidence intervals, effect sizes, degrees of freedom and P value noted<br>*Give P values as exact values whenever suitable.*
☒	☐	For Bayesian analysis, information on the choice of priors and Markov chain Monte Carlo settings
☒	☐	For hierarchical and complex designs, identification of the appropriate level for tests and full reporting of outcomes
☒	☐	Estimates of effect sizes (e.g. Cohen's d, Pearson's r), indicating how they were calculated

Our web collection on statistics for biologists contains articles on many of the points above.

Software and code

Policy information about availability of computer code

Data collection	Electrophysiology data were collected using Cheetah Data Acquisition System (Neuralynx, version 5.7.4, USA) or using Matlab custom-written code (2014b/2019a). Audiobox data was collected using Controller.
Data analysis	All analysis was performed with custom-written code in Matlab (2014b, 2019a).

For manuscripts utilizing custom algorithms or software that are central to the research but not yet described in published literature, software must be made available to editors and reviewers. We strongly encourage code deposition in a community repository (e.g. GitHub). See the Nature Research guidelines for submitting code & software for further information.

Data

Policy information about availability of data

All manuscripts must include a data availability statement. This statement should provide the following information, where applicable:

- Accession codes, unique identifiers, or web links for publicly available datasets
- A list of figures that have associated raw data
- A description of any restrictions on data availability

The data used to make the figures will be made available upon request to the corresponding author.

Field-specific reporting

Life sciences study design

All studies must disclose on these points even when the disclosure is negative.

Sample size	We used standard sample sizes used currently in the literature for auditory electrophysiology measurements.
Data exclusions	Multiunit recordings that did not show a significant response (paired t-test comparing baseline and post-sound presentation 200 ms window) were excluded from the analysis. Further exclusions for electrophysiology single measurements were only made for those data points representing outliers. For behavioral experiments, in Gap-PPI analysis, noisy trials (3 times the standard deviation of the root mean square of a 500 ms window before sound presentation) were discarded. For Audiobox experiments, a lower than 60% no-nosepoke rate during the conditioning phase resulted in removal of those animals from the experiments.
Replication	All experiments were run across several replications to ensure reproducibility. Behavioral experiments were run in larger animal cohorts, and 2 to 3 replications of each experiment were made.
Randomization	Allocation of experimental animals was performed based on the littermate genotypes. Randomly, a similar number of control and mutant animals were chosen for each litter.
Blinding	The experimenter was not blinded to the genotypes of the different mouse models. However, in electrophysiology measurements, the experimenter cannot easily influence the responses in experiments run routinely over several days. Some of our behavioral tests consisted in large groups of animals performing the experiment in an automated system, with minimal interference from the experimenter (except once weekly to clean their environment). All analysis was done using custom made code in Matlab, with fixed parameters that were applied equally to both control and mutant animals.

Reporting for specific materials, systems and methods

We require information from authors about some types of materials, experimental systems and methods used in many studies. Here, indicate whether each material, system or method listed is relevant to your study. If you are not sure if a list item applies to your research, read the appropriate section before selecting a response.

Materials & experimental systems

n/a	Involved in the study	
☐	☒	Antibodies
☒	☐	Eukaryotic cell lines
☒	☐	Palaeontology and archaeology
☐	☒	Animals and other organisms
☒	☐	Human research participants
☒	☐	Clinical data
☒	☐	Dual use research of concern

Methods

n/a	Involved in the study	
☒	☐	ChIP-seq
☒	☐	Flow cytometry
☒	☐	MRI-based neuroimaging

Antibodies

Antibodies used	anti-IBA1 (019-19741, Wako, 1:1000), anti-APP (MAB348, Millipore, 1:1000), anti-GFAP (Z0334, Dako, 1:200), anti-MAC3 (553322, BD Pharmingen, 1:400), anti-CD3 (ab11089, abcam, 1:250), rabbit anti-MBP (1:500, Dako A0623, Santa 845 Clara, U.S.A), rabbit anti-AnkyrinG (Santa Cruz, 1:100), guinea pig anti KCNQ3n (1:200, provided by Ed Cooper, Baylor College of Medicine, Houston).
Validation	IBA1: references, validation data and references available online: https://labchem-wako.fujifilm.com/us/product/detail/W01W0101-1974.html https://antibodyregistry.org/search?q=019-19741<br>APP: references, validation data and references available online: https://www.merckmillipore.com/DE/de/product/Anti-APP-A4-Antibody-a.a.-66-81-of-APP-NT-clone-22C11,MM_NF-MAB348#documentation https://antibodyregistry.org/search.php?q=AB_94882<br>GFAP: product information: https://www.agilent.com/store/en_US/Prod-Z033429-2/Z033429-2 references: https://antibodyregistry.org/search.php?q=AB_10013382<br>MAC3: references, validation data and references available online: https://www.bdbiosciences.com/us/reagents/research/antibodies-buffers/immunology-reagents/anti-mouse-antibodies/cell-surface-antigens/purified-rat-anti-mouse-cd107b-m384/p/553322 https://antibodyregistry.org/search.php?q=AB_394780<br>CD3: references, validation data and references available online: https://www.abcam.com/cd3-antibody-cd3-12-ab11089.html https://antibodyregistry.org/search?q=ab11089<br>MBP: product discontinued, no antibody datasheet available online, references: https://antibodyregistry.org/search?q=Dako%20A0623<br>For immunohistochemistry experiments, sections without primary antibody application were used as internal negative controls.

Animals and other organisms

Policy information about studies involving animals; ARRIVE guidelines recommended for reporting animal research

Laboratory animals	All mice (Mus musculus) were breed under the C57BL/6 background, both sexes were used for experiments and animals were between 6 to 12 weeks of age.
Wild animals	This study did not involve wild animals.
Field-collected samples	This study did not involve samples collected in the field.
Ethics oversight	The experimental and surgical procedures were approved and performed in accordance with the Niedersächsisches Landesamt für Verbraucherschutz und Lebensmittelsicherheit (license number 33.19-42502-04-16/2337 and 33.19-42502-04-14/1465).

Note that full information on the approval of the study protocol must also be provided in the manuscript.
